# Glutamine antagonist DRP-104 suppresses tumor growth and enhances response to checkpoint blockade in *KEAP1* mutant lung cancer

**DOI:** 10.1126/sciadv.adm9859

**Published:** 2024-03-27

**Authors:** Ray Pillai, Sarah E. LeBoeuf, Yuan Hao, Connie New, Jenna L. E. Blum, Ali Rashidfarrokhi, Shih Ming Huang, Christian Bahamon, Warren L. Wu, Burcu Karadal-Ferrena, Alberto Herrera, Ellie Ivanova, Michael Cross, Jozef P. Bossowski, Hongyu Ding, Makiko Hayashi, Sahith Rajalingam, Triantafyllia Karakousi, Volkan I. Sayin, Kamal M. Khanna, Kwok-Kin Wong, Robert Wild, Aristotelis Tsirigos, John T. Poirier, Charles M. Rudin, Shawn M. Davidson, Sergei B. Koralov, Thales Papagiannakopoulos

**Affiliations:** ^1^ Department of Pathology, New York University Grossman School of Medicine, New York, NY 10016, USA.; ^2^ Division of Pulmonary and Critical Care Medicine, Department of Medicine, VA New York Harbor Healthcare System, New York, NY 10016, USA.; ^3^ Division of Pulmonary, Critical Care, and Sleep Medicine, Department of Medicine, New York University Grossman School of Medicine, New York, NY 10016, USA.; ^4^ Applied Bioinformatics Laboratories, New York University Langone Health, New York, NY 10016, USA.; ^5^ Laura and Isaac Perlmutter Cancer Center, New York University Langone Health, New York, NY 10016, USA.; ^6^ Departments of Biological Engineering and Biology, Massachusetts Institute of Technology, Cambridge, MA 02139, USA.; ^7^ Lewis-Sigler Institute for Integrative Genomics, Princeton University, Princeton, NJ 08544, USA.; ^8^ Division of Pulmonary and Critical Care Medicine, Department of Medicine, The Feinberg School of Medicine, Northwestern University, Chicago, IL 60611, USA.; ^9^ Institute of Clinical Sciences, Department of Surgery, Sahlgrenska Center for Cancer Research, University of Gothenburg, 41345 Gothenburg, Sweden.; ^10^ Wallenberg Centre for Molecular and Translational Medicine, University of Gothenburg, 41345 Gothenburg, Sweden.; ^11^ Department of Microbiology, New York University Langone Health, New York, NY 10016, USA.; ^12^ Dracen Pharmaceuticals Inc., San Diego, CA 92121, USA.; ^13^ Department of Medicine, Thoracic Oncology Service, Memorial Sloan Kettering Cancer Center, New York, NY 10655, USA.; ^14^ Rutgers Cancer Institute of New Jersey, New Brunswick, NJ 08901, USA.

## Abstract

Loss-of-function mutations in *KEAP1* frequently occur in lung cancer and are associated with poor prognosis and resistance to standard of care treatment, highlighting the need for the development of targeted therapies. We previously showed that *KEAP1* mutant tumors consume glutamine to support the metabolic rewiring associated with NRF2-dependent antioxidant production. Here, using preclinical patient-derived xenograft models and antigenic orthotopic lung cancer models, we show that the glutamine antagonist prodrug DRP-104 impairs the growth of *KEAP1* mutant tumors. We find that DRP-104 suppresses *KEAP1* mutant tumors by inhibiting glutamine-dependent nucleotide synthesis and promoting antitumor T cell responses. Using multimodal single-cell sequencing and ex vivo functional assays, we demonstrate that DRP-104 reverses T cell exhaustion, decreases T_regs_, and enhances the function of CD4 and CD8 T cells, culminating in an improved response to anti-PD1 therapy. Our preclinical findings provide compelling evidence that DRP-104, currently in clinical trials, offers a promising therapeutic approach for treating patients with *KEAP1* mutant lung cancer.

## Teaser

DRP-104 enhances immunotherapy responses in *KEAP1* mutant lung cancer by suppressing T cell exhaustion and decreasing T_regs_.

## INTRODUCTION

Somatic mutations found in cancers play an important role in promoting tumorigenesis by driving multiple hallmarks of cancer including metabolic rewiring and immune evasion (*1*–*4*). As a result, precision medicine–based therapies that directly target driver mutations or downstream dependencies have shown great promise (*5*–*10*). Loss-of-function Kelch-like ECH-associated protein 1 (*KEAP1*) mutations or gain-of-function nuclear factor erythroid 2-related factor 2 (*NFE2L2*; also known as *NRF2*) mutations are found in ~20% of lung adenocarcinoma (LUAD) and in ~30% of lung squamous cell carcinoma (LUSC) (*11*, *12*). LUAD and LUSC are the two major histologic subtypes of non–small cell lung cancer (NSCLC). KEAP1 is a negative regulator of NRF2 (*13*–*17*), a key transcription factor that governs the cell’s antioxidant response (*11*, *12*, *14*, *17*). In LUAD, *KEAP1* mutant tumors respond poorly to checkpoint blockade (*18*–*20*) and are more resistant to KRAS^G12C^ inhibitors (*10*, *21*, *22*). Unfortunately, there are no clinically approved therapies that specifically target *KEAP1* mutant LUAD.

Multiple preclinical studies have shown that *KEAP1* loss leads to NRF2 activation, which promotes LUAD progression and metastasis (*2*, *3*, *23*–*35*). Our group previously demonstrated that NRF2 activation by *KEAP1* loss rewires cellular metabolism and generates a vulnerability that can be targeted by glutaminase (GLS1) inhibition with CB-839 (*2*, *36*). Furthermore, additional studies established that NRF2 activation in multiple cancers drives a glutamine dependency (*2*, *37*). However, CB-839 showed limited efficacy in a clinical trial that enrolled patients with *KEAP1* mutant lung cancer possibly because this compound targets only one of many glutamine-dependent reactions that are essential for cancer growth. Therefore, the development of other therapies to target *KEAP1* mutant NSCLC remains a pressing clinical issue.

6-Diazo-5-oxo-l-norleucine (DON), a glutamine antagonist, previously showed promising antitumor effects (*38*). However, clinical utility was limited due to adverse effects (*39*–*42*). Recently, DRP-104 (sirpiglenastat), a prodrug of DON, was developed as a new cancer agent with reduced toxicity as its activation is dependent on two enzymatic reactions occurring in the tumor microenvironment (*43*, *44*). However, previous studies evaluating the efficacy of DRP-104 were restricted to subcutaneous tumor mouse models without defined tumor genetics (*43*, *44*), and conducted in the absence of the native lung microenvironment where antitumor immune responses and therapeutic responses can be drastically different (*45*). On the basis of our earlier work (*2*, *46*), we hypothesize that *KEAP1* mutant lung tumors would be highly sensitive to DRP-104 due to the increased glutamine dependency of these tumors. Here, we use an antigenic orthotopic lung cancer mouse model and patient-derived xenografts (PDXs) to investigate the impact of DRP-104 on *KEAP1* mutant lung tumor growth. We found that in these preclinical models, *KEAP1* mutant tumors are highly sensitive to DRP-104, as compared to *KEAP1* wild-type (WT) tumors. Using a systematic metabolomics approach, we found that the cell-intrinsic sensitivity of *KEAP1* mutant tumors to DRP-104 is primarily mediated by inhibition of nucleotide synthesis. Furthermore, after a comprehensive immune analysis using flow cytometry and multimodal single-cell sequencing [expanded cellular indexing of transcriptomes and epitopes by sequencing (ExCITE-seq)] in our orthotopic mouse model, we found that DRP-104 reduces exhausted CD4 and CD8 T cell populations, enhances T cell cytokine production, and augments the response to anti-PD1 checkpoint inhibitor therapy in *Keap1* mutant tumors. In summary, our research establishes a convincing mechanistic rationale for the ongoing clinical trial combining DRP-104 with checkpoint blockade in patients with *KEAP1* mutant LUAD (NCT04471415).

## RESULTS

### *KEAP1* mutant tumor growth is impaired by DRP-104

**Fig. 1.**
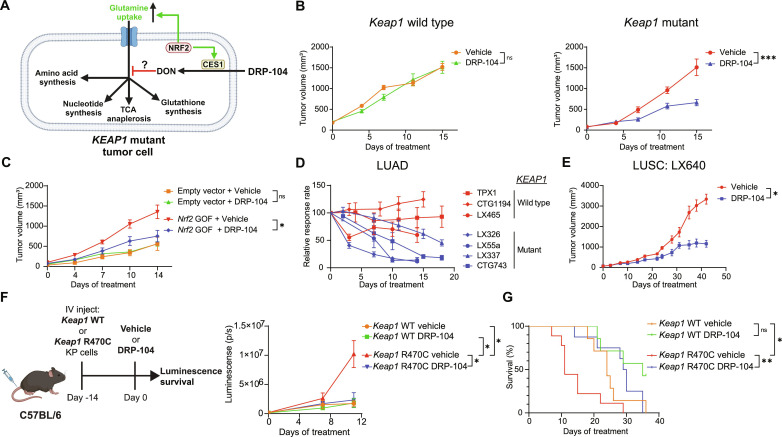
*KEAP1* mutant tumors are sensitive to DRP-104 in vivo. (**A**) *KEAP1* mutant tumors have enhanced NRF2 activity leading to metabolic rewiring and enhanced glutamine uptake, potentially sensitizing them to DRP-104, the prodrug of DON. CES1, carboxylesterase 1. (**B**) *Keap1* WT or mutant *Kras*^G12D/+^ *p53*^−/−^ (KP) cells were subcutaneously transplanted into nude mice (*n* = 15 per group). Mice were treated with either DRP-104 (2 mg/kg) or vehicle subcutaneously 5 days on, 2 days off. (**C**) KP cells were transduced with an *Nrf2* gain-of-function (GOF) vector or empty vector control and were subcutaneously transplanted into nude mice (*n* = 7 to 8 per group), and mice were treated with either DRP-104 (3 mg/kg) or vehicle control. (**D**) Lung adenocarcinoma patient-derived xenografts (PDXs) were implanted into NSG mice. Mice were treated with either vehicle control or DRP-104 (3 mg/kg), and tumors were measured over time. Relative response rate (tumor volume/average vehicle volume × 100%) over time is plotted. *KEAP1* WT and mutant PDXs are labeled. (**E**) Growth kinetics of the lung squamous cell carcinoma PDX LX640 treated with DRP-104 (3 mg/kg) or vehicle control (*n* = 8 per group). (**F** and **G**) Schematic of orthotopic transplant lung cancer model. *Keap1* WT or *Keap1* R470C mutant KP cell lines expressing luciferase were intravenously (IV) injected into C57BL/6 mice on day 0. On day 14, lung luminescence (p/s, photons/second) was measured and mice were randomized into treatment groups (seven to nine mice per group) with either DRP-104 (3 mg/kg) or vehicle control. Tumor growth kinetics based on luminescence was measured (F), and survival data (G) are shown. Data are plotted as mean with SEM. For statistical analysis, two-way analysis of variance (ANOVA) was used for growth kinetics and log-rank test was used for survival. ns, not significant; *P < 0.05, **P < 0.01, ***P < 0.001, ****P < 0.0001.

*KEAP1* mutant tumors have an increased glutamine dependency to support the metabolic rewiring associated with activation of NRF2 (Fig. 1A) (*2*). In addition, CES1, the enzyme that activates the prodrug DRP-104 into DON (fig. S1A), is an NRF2 target (Fig. 1A) (*47*). We therefore suspected that *KEAP1* mutant tumors would be highly susceptible to the glutamine antagonist DRP-104. To determine the effect of DRP-104 on *Keap1* mutant tumors, we transplanted murine *Kras*^G12D/+^ *p53*^−/−^ *Keap1* knockout (KPK) cell lines generated by CRISPR/Cas9 editing or *Keap1* WT (KP) cell lines subcutaneously into immunodeficient mice. We observed that KPK tumors were sensitive to escalating doses of DRP-104, while KP tumors were resistant (Fig. 1B). Both cell lines were sensitive to DRP-104 and DON in vitro, with KPK cell lines being more sensitive (fig. S1B). Since DON failed in clinical trials due to its high toxicity when delivered systemically, we monitored mice for adverse effects during treatment with multiple doses of DRP-104 and observed no evidence of weight loss or toxicity (fig. S1C). Our previous work has demonstrated that loss of function of *Keap1* increases NRF2 transcriptional activity and promotes glutamine addiction in LUAD mouse models (*2*, *36*). To determine whether sensitivity of KPK cells to DRP-104 is due to this NRF2-mediated glutamine addiction, we overexpressed a gain-of-function mutant of *Nrf2*, which has a deletion in the Neh2 domain and cannot bind KEAP1 (*2*), in KP cells and measured the sensitivity of this cell line to DRP-104 in vivo. Consistent with the loss-of-function *Keap1* mutant tumors, we observed that *Nrf2* gain-of-function tumors were also sensitive to DRP-104 (Fig. 1C). This finding demonstrates that NRF2 activation, and likely the subsequent glutamine addiction it induces, sensitizes cells to DRP-104.

*KEAP1* mutations frequently co-occur with serine/threonine kinase 11 [*STK11*, also known as liver kinase B1 (*LKB1*)] mutations in human LUAD, and the co-occurrence of these mutations is associated with resistance to therapies through unknown mechanisms (*19*). To verify that the sensitivity of KPK tumors to DRP-104 is retained with loss-of-function mutations in *Stk11*, we generated KPK cells with loss of *Stk11.* When transplanted subcutaneously, these *Keap1/Stk11* mutant tumors were indeed also sensitive to DRP-104, thereby demonstrating that *Stk11* mutations do not induce resistance to DRP-104 (fig. S1D). Furthermore, to investigate whether *Stk11* mutation status has an effect on DRP-104 sensitivity in the setting of *Keap1* WT tumors, we treated *Stk11* mutant KP tumors and found that DRP-104 still had no antitumor effect (fig. S1E).

Given the substantial degree of genetic heterogeneity in patient tumors, which may lead to drug resistance, we wanted to ascertain the effectiveness of DRP-104 in multiple genetically defined PDX models of NSCLC. To accomplish this, we tested seven LUAD PDX lines (three *KEAP1* WT and four *KEAP1* mutant), each with different co-occurring mutations (fig. S2A). *KEAP1* WT PDX models did not show a significant response to DRP-104 (TPX1, CTG1194, and LX465) (Fig. 1D and fig. S2B). Remarkably, all four *KEAP1* mutant PDXs (LX326, LX55a, LX337, and CTG743) demonstrated a robust response to DRP-104 (Fig. 1D and fig. S2, C and D). Furthermore, DRP-104 maintained long-term suppression of the *KEAP1* mutant PDX CTG743, as demonstrated by significant tumor regression followed by sustained maintenance of tumor growth inhibition during an extended dosing period of 50 days without evidence of resistance (fig. S2D). Withdrawal of the drug results in resumption of tumor growth in this PDX, suggesting that, in an immunodeficient host, sustained drug administration is required to maintain efficacy (fig. S2D). Since NRF2 activation by either loss-of-function mutation of *KEAP1* or gain-of-function mutation of NRF2 is observed in approximately 30% of LUSC, the other major subtype of NSCLC (*11*), we also tested the therapeutic efficacy of DRP-104 in a LUSC PDX model (LX640) with *KEAP1* mutation. Consistent with the LUAD PDXs, we observed that DRP-104 suppressed the growth of *KEAP1* mutant LUSC (Fig. 1E).

Previous work has demonstrated that treatment responses in the lung can markedly differ from those in subcutaneous tissue, a discrepancy partly attributable to antitumor immune responses (*45*). In addition, inhibiting glutamine metabolism can have profound effects on immune cell function (*48*, *49*). With this in mind, we sought to evaluate the efficacy of DRP-104 using an antigenic orthotopic lung transplant model that we have recently established (*50*). With this model, we have demonstrated that KP tumor cell lines expressing a *Keap1* loss-of-function point mutation (R470C) grow faster in the lung than those expressing WT *Keap1*, primarily by suppressing CD8 T cell antitumor surveillance (*50*). Further using this model, we transplanted *Keap1* R470C mutant or *Keap1* WT KP tumor cells, each expressing luciferase, and continuously tracked the lung tumor burden through bioluminescence imaging (Fig. 1F). Upon engraftment of tumors in the lung, mice were treated with DRP-104 or vehicle. Consistent with our subcutaneous in vivo model (Fig. 1B), DRP-104 impaired the growth of *Keap1* R470C mutant lung tumors (Fig. 1F) as well as significantly increased the median survival of mice with *Keap1* R470C mutant lung tumors from 11 days to 35 days (Fig. 1G). Overall, using both human and murine tumor models in immunodeficient and immunocompetent mice, we demonstrate that DRP-104 effectively inhibits the growth of *KEAP1* mutant lung tumors and, in some cases, results in tumor regression.

### DRP-104 impairs tumor proliferation by inhibition of nucleotide synthesis

**Fig. 2.**
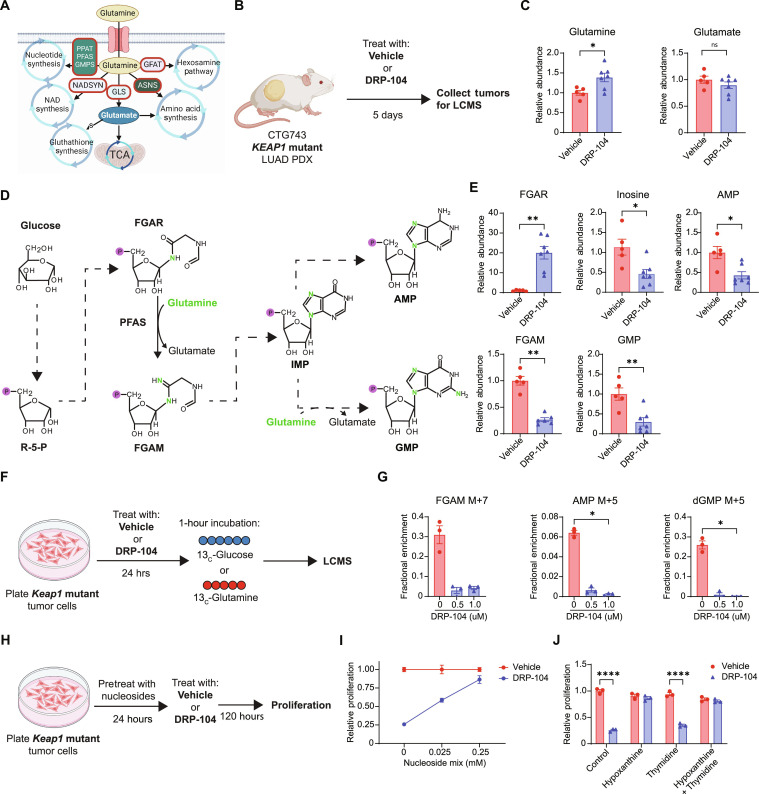
DRP-104 targets nucleotide metabolism in *KEAP1* mutant tumors. (**A**) Overview of glutamine-dependent pathways. (**B**) Schematic of in vivo metabolomics. After implantation of the CTG743 (*Keap1* mutant) PDX into NSG mice, treatment with DRP-104 (3 mg/kg) or vehicle control (*n* = 5 to 7 per group) was initiated. After 5 days of treatment, tumors were collected for liquid chromatography mass spectrometry (LCMS). (**C**) Relative abundance of glutamine and glutamate as measured by LCMS from experiment in (B). (**D**) Outline of purine biosynthesis where glucose is used to generate ribose-5-phopshate (R-5-P), FGAR, FGAM, IMP, AMP, and GMP. (**E**) Relative abundance of FGAR, FGAM, inosine, AMP, and GMP as measured by LCMS in *KEAP1* mutant PDX tumors after treatment with DRP-104 (3 mg/kg). (**F**) *Keap1* mutant tumor cells were treated with DRP-104 (0, 0.5, or 1 μM) (*n* = 3 per group). After 24 hours, cells were incubated with either labeled ^13^C-glucose or ^13^C-glutamine for 1 hour and then cells were collected for LCMS. (**G**) Fractional enrichment of FGAM, AMP, and dGMP for experiment outlined in (F). (**H**) *Keap1* mutant tumor cells were pretreated with the nucleosides cytidine, hypoxanthine, uridine, thymidine, guanosine, and adenosine (0 to 0.25 mM) for 24 hours and then treated with DRP-104 (2 μM) or control medium for 120 hours (*n* = 3 per group). Proliferation was measured by crystal violet. (**I**) Plot of nucleoside mix concentration versus relative proliferation of *Keap1* mutant tumor cells treated with DRP-104 normalized to control cells. (**J**) Relative proliferation of DRP-104 or vehicle-treated *Keap1* mutant tumor cells after addition of either hypoxanthine or thymidine or both. Statistical analysis was done by either Mann-Whitney test, Kruskal-Wallis test with Dunn’s multiple-comparisons test, or two-way ANOVA. *P < 0.05, **P < 0.01, ***P < 0.001, ****P < 0.0001.

Glutamine is used in multiple biosynthetic pathways including nucleotide synthesis, nicotinamide adenine dinucleotide (NAD) synthesis, glutathione production, hexosamine pathway, and amino acid synthesis, as well as for replenishing tricarboxylic acid (TCA) intermediates via α-ketoglutarate (Fig. 2A) (*51*). We hypothesized that DRP-104 has superior efficacy against *KEAP1* mutant tumors compared to previous selective glutaminase inhibitors due to its ability to target multiple glutamine-dependent metabolic pathways. To probe the metabolic pathways affected by DRP-104, we performed in vivo metabolomics using our *KEAP1* mutant (CTG743) PDX model and harvested tumors after 5 days of treatment with DRP-104 or vehicle (Fig. 2B). We have previously observed that CB-839 primarily affected *KEAP1* mutant tumors through reduction of intracellular glutamate via inhibition of GLS1 (*36*). However, our liquid chromatography mass spectrometry (LCMS) analysis demonstrated that while DRP-104 levels did increase glutamine levels, DRP-104 treatment did not significantly reduce glutamate levels, suggesting that inhibition of glutaminolysis is not a major effect of DRP-104 in vivo (Fig. 2C) and acts through mechanisms distinct from CB-839.

We then systematically investigated other metabolic pathways using glutamine (Fig. 2A) to identify the vulnerability of *KEAP1* mutant tumors to DRP-104. Given that nucleotide synthesis plays a pivotal role in cell proliferation, we next focused on the synthesis of purines and pyrimidines. For purine synthesis, the generation of inosine monophosphate (IMP), the purine precursor for adenosine monophosphate (AMP) and guanosine monophosphate (GMP), requires glutamine as a nitrogen source (*52*) to generate formylglycinamidine ribonucleotide (FGAM) from formylglycinamide ribonucleotide (FGAR) and this reaction is mediated by the enzyme phosphoribosylformylglycinamidine synthase (PFAS) (Fig. 2D). We found that FGAR abundance was significantly increased, while FGAM was significantly reduced, in tumors upon DRP-104 treatment, suggesting that the activity of PFAS was inhibited (Fig. 2E). Consistent with an inhibition of the purine synthesis pathway, we found that inosine, AMP, and GMP levels were both significantly reduced following DRP-104 treatment (Fig. 2E). We then examined pyrimidine biosynthesis, which uses glutamine as a substrate for synthesis of the pyrimidine ring (fig. S3A). Orotate, an intermediate in pyrimidine synthesis, and downstream metabolites uridine monophosphate (UMP) and deoxythymidine monophosphate (dTMP) were significantly reduced following DRP-104 treatment (fig. S3B). CTP was not reduced after treatment with DRP-104 (fig. S3B), despite requiring glutamine for the generation of CTP from uridine triphosphate (UTP). We next examined other glutamine-dependent pathways beyond nucleotide synthesis. Glutamine is used as a substrate in the hexosamine pathway to synthesize uridine diphosphate (UDP)–*N*-acetyl-glucosamine and is also required for the generation of asparagine, aspartate, and glutathione (Fig. 2A). Our LCMS analysis demonstrated that UDP–*N*-acetyl-glucosamine and asparagine levels were unchanged, but aspartate and glutathione were significantly reduced by treatment with DRP-104 (fig. S3C).

To identify which synthetic reactions were affected by DRP-104, we performed in vitro metabolic tracing by labeling DRP-104–treated KPK tumor cells with either ^13^C-glutamine or ^13^C-glucose for 1 hour (Fig. 2F). *KEAP1* mutant tumors replenish intracellular glutamate via glutaminolysis mediated by the enzyme GLS1 (fig. S3D). Our tracing analysis demonstrated that DRP-104 did not have any significant effect on glutamate M+5 fractional labeling by ^13^C-glutamine (fig. S3D), further suggesting that DRP-104 does not markedly affect glutaminolysis. We chose to focus on the labeling of nucleotide synthesis intermediates given the alteration in purine and pyrimidine synthesis intermediates induced by DRP-104 (Fig. 2E and fig. S3B). First, we looked at labeling of intermediates of purine synthesis by ^13^C-glucose (fig. S3E) and found a trend toward a reduction in the FGAM M+7 fraction (Fig. 2G). In addition, there was a decrease in labeled AMP M+5 and dGMP M+5 fraction (Fig. 2G), demonstrating that DRP-104 impairs the biosynthetic reactions for these metabolites. Coinciding with this, we found a reduction of FGAM and a trend toward reduced levels of AMP and dGMP after 24 hours of DRP-104 treatment (fig. S3F). Similarly, when examining labeled pyrimidines, we found that DRP-104 reduced the labeling of UMP, dTMP, and CTP (fig. S3G), suggesting that DRP-104 also impairs the synthesis of pyrimidines. Consistent with the lack of changes in glutamate levels, we also did not see any reduction in labeling of TCA intermediates such as α-ketoglutarate, succinate, and fumarate (fig. S3H).

To determine whether impaired nucleotide synthesis was responsible for the reduction in tumor growth induced by DRP-104, we investigated whether the addition of nucleosides could restore the proliferation in treated *Keap1* mutant tumor cells. We pretreated KPK tumor cells in vitro with a mix of nucleosides (cytidine, thymidine, hypoxanthine, uridine, guanosine, and adenosine). After 24 hours of nucleoside pretreatment, we administered DRP-104 to the KPK cells and measured their proliferation 5 days later (Fig. 2H). Remarkably, the proliferation of DRP-104–treated KPK cells improved drastically in a dose-dependent manner with increasing nucleoside concentrations (Fig. 2I). To distinguish the necessity of purines from pyrimidines in DRP-104–treated cells, we pretreated KPK cells in vitro with either hypoxanthine, thymidine, or both. Addition of the purine hypoxanthine, but not the pyrimidine thymidine, rescued the proliferation of DRP-104–treated *Keap1* mutant tumor cells (Fig. 2J), demonstrating that while both purine and pyrimidine synthesis pathways are inhibited with DRP-104, the deficit in purines drives the reduced proliferation observed.

To comprehensively evaluate the role of other metabolic deficiencies induced by DRP-104, we performed additional metabolite rescues. While loss-of-function mutations in *KEAP1* result in increased expression of antioxidant pathways, our previous work has established that the sensitivity to glutaminase inhibition is not due to increased oxidative stress (*36*). Rather, CB-839 suppresses tumor growth by reducing intracellular glutamate stores required for TCA cycle anaplerosis (*36*, *53*) and amino acid synthesis—a suppression reversible by either glutamate supplementation or blocking glutamate export through the transporter xCT using erastin (*36*). Contrary to the effect seen with CB-839, addition of glutamate or erastin had no impact on cell proliferation of DRP-104–treated KPK cells (fig. S3I), demonstrating that DRP-104’s effect is not mediated through intracellular glutamate depletion. Supplementation with cell-permeable α-ketoglutarate [dimethyl 2-oxoglutarate (DMG)] or pyruvate also did not improve cell proliferation, suggesting that DRP-104 does not induce deficiencies in TCA cycle intermediates (fig. S3I). Finally, treatment with the aspartate or the antioxidant Trolox also failed to rescue DRP-104–treated cells despite having reduced aspartate and glutathione levels (fig. S3, C and I). We therefore concluded that while DRP-104 induces multiple metabolic deficiencies, its primary mechanism of impairing the proliferation of *KEAP1* mutant tumors is through inhibition of purine synthesis.

### DRP-104 modulates antitumor T cell responses and augments checkpoint blockade efficacy

**Fig. 3.**
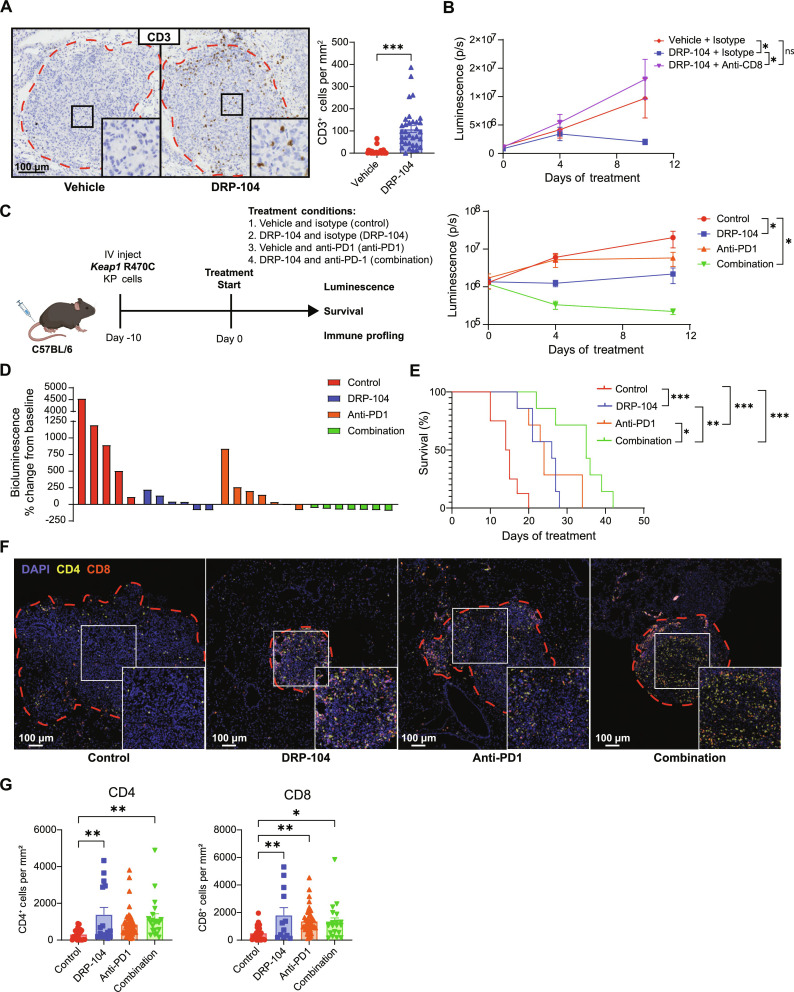
DRP-104 augments T cell infiltration and increases response rates to anti-PD1 therapy. (**A**) Immunohistochemistry staining for CD3 of mouse lungs with *Keap1* R470C KP tumors treated with either DRP-104 or vehicle control. Intratumoral CD3 quantification is shown for individual tumors. (**B**) *Keap1* R470C mutant KP tumor cells were intravenously injected into C57BL/6 mice. After 10 days, mice were treated with either anti-CD8 or isotype control (150 μg intraperitoneally twice a week) and DRP-104 or vehicle control (*n* = 4 to 6 per group). Lung tumor burden as measured by luminescence is displayed. (**C** and **D**) *Keap1* R470C mutant KP tumor cells were intravenously injected into C57BL/6 mice. After 10 days, mice were randomized into treatment conditions displayed in the schematic. Tumor burden was measured over time by luminescence (*n* = 5 to 7 per group) (C), and waterfall plot showing bioluminescence signal at day 11 relative to signal at treatment initiation is shown (D). (**E**) Survival of mice shown from experiment outlined in (C). (**F** and **G**) Multi-color immunofluorescent staining of *Keap1* R470C lung tumors (F) after 5 days of treatment with DRP-104 (+ isotype), anti-PD1 (+ vehicle), the combination of both, or controls (vehicle + isotype control). Quantification of CD4 (yellow) and CD8 (red) intratumoral T cell populations is shown for individual tumors (G). Data were analyzed by one-way ANOVA and Tukey’s multiple-comparison testing or log-rank test. *P < 0.05, **P < 0.01, ***P < 0.001, ****P < 0.0001.

The function of T cells is heavily influenced by nutrient availability. Activated T cells are highly proliferative and use numerous metabolites including glucose, asparagine, and serine (*54*–*60*). CD8 T cell function is impaired in glutamine-depleted conditions (*61*, *62*). Additionally, differentiation of CD4 T cells into various subsets, including T helper 1 (T_H_1), T_H_17, and T regulatory cells (T_regs_), is modulated by the levels of glutamine and glutamate (*49*). Furthermore, previous work has suggested that the efficacy of DON in vivo is partially mediated by the enhancement of CD8 T cell function (*48*). Recently, despite efficacy in preclinical models (*2*, *63*, *64*), blockade of GLS1 with CB-839 had failed to show efficacy in clinical trials. One possible explanation is that the beneficial effect of CB-839 on *KEAP1* mutant tumors may be offset by potential negative effects on T cell function (*61*). We therefore thought that it was necessary to evaluate the impact of DRP-104 on the immune microenvironment of *Keap1* mutant lung tumors. We first sought to determine whether T cell infiltration is altered by DRP-104 in *Keap1* mutant lung tumors. To investigate this, we performed immunohistochemistry for CD3 to quantify T cell infiltration of end-stage *Keap1* R470C mutant lung tumors. In vehicle-treated mice, *Keap1* mutant tumors demonstrated immune exclusion with very low intratumoral T cell infiltration (Fig. 3A). However, DRP-104 significantly increased the infiltration of T cells into tumors (Fig. 3A). These findings suggest that one of the mechanisms by which DRP-104 might suppress tumor growth in vivo is by enhancing antitumor T cell responses against *Keap1* mutant tumors.

We previously showed that CD8 T cell depletion had no impact on *Keap1* mutant tumor growth using this model (*50*). To determine whether T cells were now generating antitumor responses in the context of the glutamine antagonist, we evaluated the effect of CD8 T cell depletion on DRP-104–treated *Keap1* mutant tumors. To do this, we injected mice with *Keap1* R470C mutant cells via tail vein injection. After tumors had successfully engrafted in the lung, we treated mice with vehicle and isotype control, DRP-104 with isotype control, or DRP-104 with anti-CD8–depleting antibody. In contrast to our work where anti-CD8 antibodies had no effect on *Keap1* mutant tumor growth (*50*), CD8 T cell depletion accelerated the growth of *Keap1* R470C mutant tumors in the presence of DRP-104 (Fig. 3B), suggesting that DRP-104 can activate antitumor T cell responses against *Keap1* mutant tumors.

Patients with *KEAP1* mutant LUAD are known to have poor responses to checkpoint blockade (*19*, *65*), and our previous work has similarly demonstrated that this also holds true in our *Keap1* mutant orthotopic tumor mouse model (*50*). Given our findings that DRP-104 enhances T cell infiltration and induces T cell–mediated antitumor responses (Fig. 3, A and B), we next asked whether DRP-104 can augment antitumor responses when combined with immunotherapy, the standard of care for advanced LUAD. Following the injection of *Keap1* R470C mutant KP cells, we administered either DRP-104 or a vehicle control to the mice, along with either anti-PD1 or isotype control antibody (Fig. 3C). Lung tumor burden was then monitored via bioluminescence. Consistent with previous results, tumor growth was impaired with DRP-104 (Fig. 3C). While anti-PD1 alone dampened tumor growth, combination of anti-PD1 with DRP-104 significantly reduced tumor growth (Fig. 3C). After 11 days of treatment, while DRP-104 generally slowed tumor growth, combination therapy induced regressions in all treated mice at this time point (Fig. 3, C and D). In an independent cohort, we found that combination therapy markedly increased survival of mice with *Keap1* mutant lung tumors (with a median survival difference exceeding 20 days) (Fig. 3E).

Given the increased infiltration of T cells and their functional importance in suppression of tumor growth in response to DRP-104, we next evaluated which T cell subsets were affected in the tumor microenvironment of animals treated with DRP-104 alone and in combination with anti-PD1. We collected tumor-bearing lungs after 5 days of treatment to minimize differences in immune infiltration associated with disparities in tumor burden. Using multi-immunofluorescence, we stained for CD4 and CD8 T cells and quantified the intratumoral populations (Fig. 3F). We found that both CD4 and CD8 populations were increased with DRP-104–treated animals compared to the control group (Fig. 3G). Anti-PD1 monotherapy resulted in an increase in CD8 infiltration without significantly altering CD4 infiltration (Fig. 3G). While the combination of DRP-104 and anti-PD1 increased the infiltration of CD4 and CD8 T cells compared to the control arm, this infiltration was not significantly different than the DRP-104 single treatment condition (Fig. 3G). The fact that combination therapy significantly improved survival compared to monotherapy (Fig. 3E) despite comparable T cell infiltration among the treatment arms (Fig. 3, F and G) raises the possibility that the functionality of T cells is enhanced with the combination of DRP-104 with anti-PD1 rather than simply increasing the number of T cells. Our findings overall demonstrate that DRP-104 exerts tumor-intrinsic effects by suppressing glutamine-dependent metabolism while also promoting antitumor T cell responses, thereby enhancing the efficacy of checkpoint blockade.

### Multimodal sequencing identifies T cell populations altered by DRP-104

**Fig. 4.**
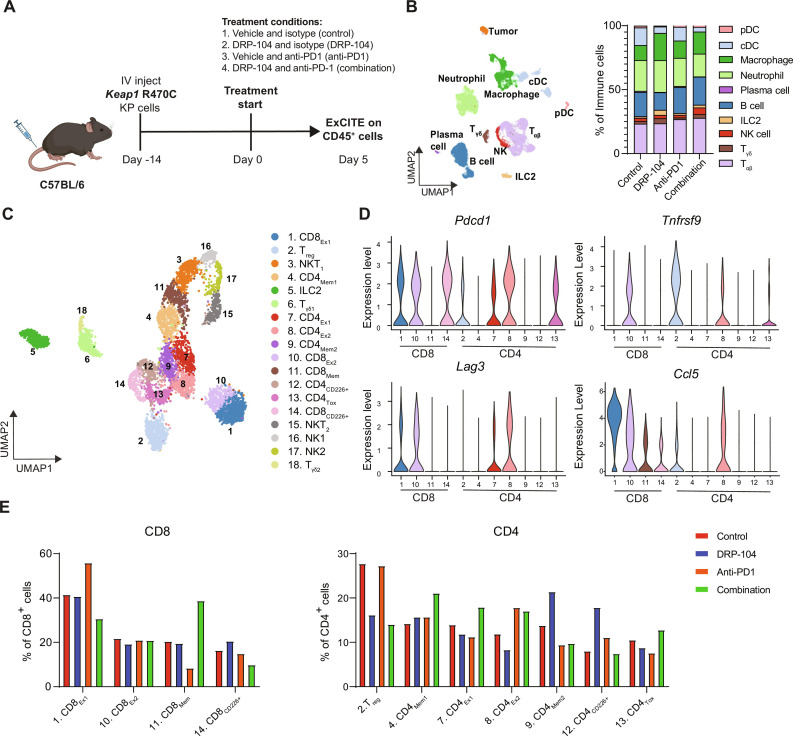
ExCITE-seq identifies transcriptional changes in T cell populations with DRP-104 and anti-PD1 therapy. (**A**) Schematic of experimental design of acquisition of samples for ExCITE-seq. Fourteen days after injection of *Keap1* R470C mutant KP cells, mice were treated with DRP-104 (3 mg/kg) or vehicle and anti-PD1 (200 μg intraperitoneally every other day) or isotype control. Tumor-bearing lungs were digested after 5 days of treatment, and extravascular CD45^+^ cells and tumor cells were sorted for analysis by ExCITE-seq (*n* = 2 per group). (**B**) UMAP showing clustering of cell populations with quantification of immune subpopulations. (**C**) Subcluster showing T cell, NKT cell, NK cell, and ILC populations, with subclusters labeled. (**D**) Violin plots showing expression of *Pdcd1*, *Tnfrsf9*, *Lag3*, and *Ccl5* by T cell clusters shown in (C). (**E**) Quantification of T cell subclusters stratified by treatment condition and normalized to total CD8 or CD4 T cells.

Intratumoral T cells are a heterogeneous population composed of effector, exhausted, and memory CD8s, along with various CD4 T cell subsets such as T_H_1, T_H_17, and T_regs_. Given the general diversity of T cell populations and our observation that DRP-104 may alter the functionality of T cells, we chose to comprehensively examine the immune microenvironment of *Keap1* mutant tumors using the multimodal single-cell platform ExCITE-seq (*66*, *67*) to identify immune populations that are affected by glutamine antagonism. ExCITE-seq uses oligo-tagged antibodies to simultaneously provide surface epitope information along with gene expression at a single-cell resolution (*66*–*68*). We treated mice with *Keap1* R470C mutant lung tumors with DRP-104 and/or anti-PD1 and harvested whole lungs for single-cell analysis after 5 days of treatment (Fig. 4A), a time point where tumor burden differences among treatment arms are minimal (Fig. 3C). Lungs were digested, and noncirculating CD45^+^ immune cells and tumor populations were sorted out and subsequently analyzed by ExCITE-seq (Fig. 4A). Our initial clustering revealed a diverse subset of immune populations including macrophages, neutrophils, B cells, T cells, and natural killer (NK) cells (Fig. 4B). In general, we observed a subtle increase in total T cell populations with combination therapy (Fig. 4B), further supporting that functional changes may contribute to the robust suppression in tumor growth by CD8 T cells opposed to T cell numbers (Fig. 3B). We focused our single-cell analysis on lymphoid populations composed of T cells, NKT cells, NK cells, and innate lymphoid cells (ILCs), with clustering of these populations shown in Fig. 4C. Using antibody-derived tags (ADTs), we were able to identify CD4 and CD8 T cell populations (fig. S4A). When looking at relative gene expression in CD4 and CD8 T cells, we observed a clear up-regulation of several genes associated with activation, such as *Cd44*, *Pdcd1*, and *Nkg7* brought about by DRP-104 and/or anti-PD1 (fig. S4B). Concurrently, we noticed a down-regulation of genes associated with naïve populations, such as *Ccr7* and *Sell* (fig. S4B).

To gain greater understanding of the diverse adaptive immune populations and transcriptional changes induced by DRP-104, we subclustered the T cell, ILC, NK, and NKT cells into 18 subclusters (Fig. 4C). Seven of these clusters were identified as CD4 T cells, and four as CD8 T cells (Fig. 4C and fig. S4A). Differential gene expression and ADT expression facilitated identification of these T cell populations (Fig. 4D and fig. S4, C and D). We identified four CD8 T cell clusters, with two of these clusters (labeled CD8_Ex1_ and CD8_Ex2_) coexpressing *Lag3* and *Pdcd1* (Fig. 4D), likely representing some degree of exhaustion (*69*–*72*). These two exhausted T cell clusters distinguish themselves, with CD8_Ex1_ displaying higher *Ccl5* expression, while CD8_Ex2_ expresses *Tnfrsf9* (Fig. 4D), another marker of exhaustion. Notably, *Ccl5*-expressing CD8 T cells have previously been implicated with a dysfunctional state and poor responses to anti-PD1 therapy in orthotopic lung cancer models (*45*). Our data demonstrate that anti-PD1 therapy expands these *Ccl5*-expressing dysfunctional CD8 T cells (CD8_Ex1_) (Fig. 4E) within the lungs of tumor-burdened mice, potentially limiting the effectiveness of anti-PD1 therapies. However, addition of DRP-104 reduces this population, possibly facilitating more effective antitumor responses (Fig. 4E). We also identified a memory CD8 cluster (CD8_Mem_) expressing Ly6C (*73*) (fig. S4E) that is preferentially expanded with combination therapy (Fig. 4E).

In a parallel manner, we looked at differences in CD4 subsets induced by DRP-104, anti-PD1, or combination therapy. Quantification of these subclusters revealed that the most markedly reduced CD4 population by DRP-104 is T_regs_ (Fig. 4E), which we have previously shown to be enriched in *Keap1* mutant tumors (*74*). A similar change in this population was observed both upon DRP-104 treatment and in response to the combination therapy. Similar to CD8 T cells, we identified two CD4 populations with coexpression of *Pdcd1* and *Lag3* expression (shown in Fig. 4D, labeled as CD4_Ex1_ and CD4_Ex2_ in Fig. 4, C and E), suggestive of an exhausted state. Both of these exhausted clusters, along with the *Tox*-expressing CD4_Tox_ cluster, were slightly decreased by DRP-104 treatment, but paradoxically expanded upon combination therapy. However, combination therapy also expanded a CD4 memory population (CD4_Mem1_), similar to memory CD8 T cells (CD8_Mem_) (Fig. 4E). In summary, our single-cell analysis demonstrates that combining DRP-104 with anti-PD1 may alter the functionality and transcriptional state of *Keap1* mutant tumor–infiltrating CD4 and CD8 T cell populations, driving them from an exhausted program toward a more functional effector/memory state.

### DRP-104 enhances the effector function of CD4 and CD8 T cells

**Fig. 5.**
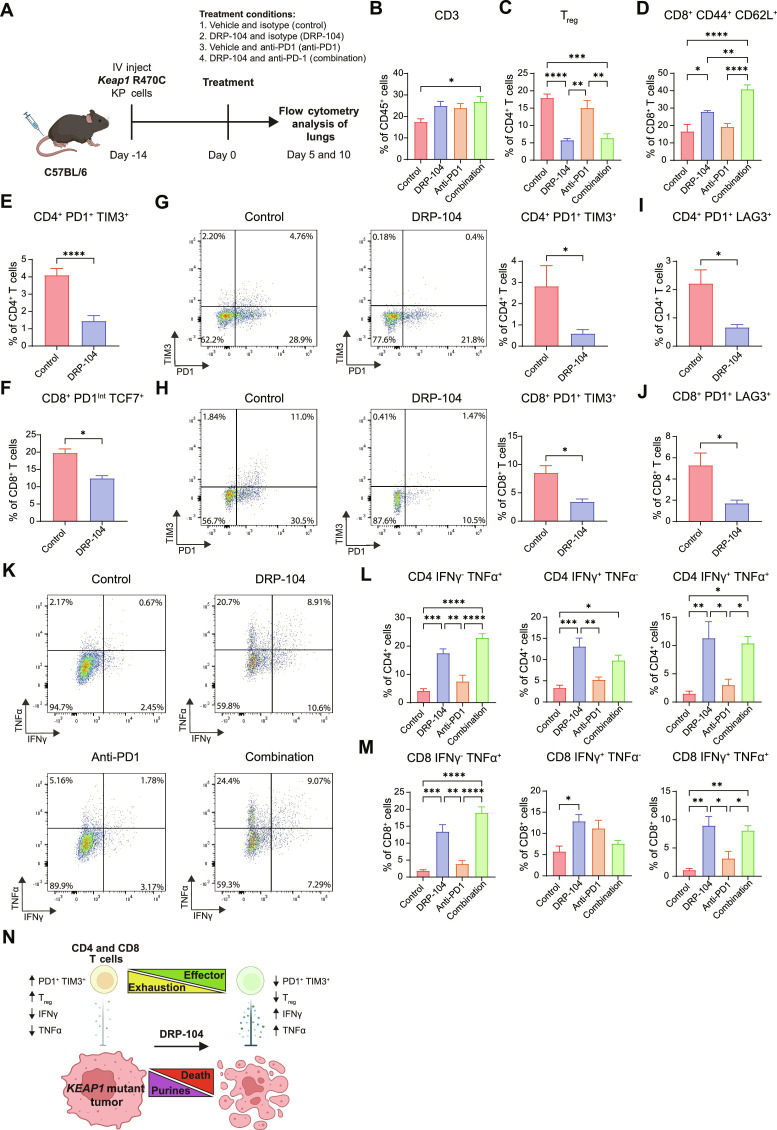
DRP-104 reduces T cell exhaustion and enhances effector T cell function in vivo. (**A**) Schematic of experimental design. *Keap1* R470C mutant KP lines were injected intravenously into C57BL/6 mice. Fourteen days after injection, treatment with DRP-104 (3 mg/kg) or vehicle and anti-PD1 (200 μg intraperitoneally three times a week) or isotype control was initiated. Lungs were collected from tumor-bearing mice either 5 or 10 days after treatment initiation and analyzed by flow cytometry. (**B** to **F**) Flow cytometry quantification of (B) CD3 T cells, (C) T_regs_ (CD4^+^ FoxP3^+^ CD25^+^), (D) CD8^+^ CD44^+^ CD62L^+^ (central memory CD8 T cells), (E) CD4^+^ PD1^+^ TIM3^+^, and (F) CD8^+^ PD1 intermediate TCF7^+^ populations after 5 days of treatment (*n* = 5 per group). (**G** and **H**) Flow cytometry analysis of PD1^+^ TIM3^+^ populations for (G) CD4 T cells and (H) CD8 T cells with representative gating after 10 days of treatment with DRP-104 (*n* = 3 to 6 per group). (**I** and **J**) Flow cytometry quantification of PD1^+^ LAG3^+^ for (I) CD4 T cells and (J) CD8 T cells (*n* = 3 to 6 per group) after 10 days of treatment with DRP-104. (**K** to **M**) Representative gating (K) and flow cytometry quantification of IFNγ and TNFα expression for PMA/ionomycin-stimulated (L) CD4 T cells and (M) CD8 T cells after 5 days of treatment with DRP-104 or vehicle and/or anti-PD1 or isotype control (*n* = 5 per group). (**N**) Overview of effect of DRP-104 on *KEAP1* mutant tumors and T cells. Data were analyzed by either Mann-Whitney test or one-way ANOVA and Tukey’s multiple-comparison testing. *P < 0.05, **P < 0.01, ***P < 0.001, ****P < 0.0001.

Our ExCITE-seq data revealed that DRP-104 and anti-PD1 therapy had a marked effect on specific T cell populations, including T_regs_, and on the distribution of memory versus exhausted T cell subsets. We then aimed to corroborate these findings and further characterize the functionality of T cells within the tumor microenvironment of *Keap1* mutant lung tumors treated with DRP-104. We performed multi-color flow cytometry on *Keap1* R470C mutant KP tumor-bearing lungs (gating shown in fig. S5A) after 5 and 10 days of treatment with DRP-104, anti-PD1, or the combination of both (Fig. 5A). After 5 days of treatment, we observed a modest increase in CD3 T cells following combination therapy with anti-PD1 and DRP-104, in comparison to untreated controls (Fig. 5B). Despite not being statistically significant, this increase in T cell population is likely driven by small increases in CD4 and CD8 T cell subsets (fig. S5B). Consistent with our ExCITE-seq data, DRP-104 profoundly decreased the proportion of T_regs_ (Figs. 4E and 5C). Furthermore, we observed an increase in CD8 central memory populations (identified by expression of CD44^+^ and CD62L^+^) following DRP-104 treatment, which was further augmented with addition of anti-PD1 (Fig. 5D).

We next shifted our focus on evaluating T cell exhaustion since our ExCITE-seq analysis suggested that DRP-104 reduced T cell–exhausted subsets (Fig. 4E). On the basis of several studies (*69*, *70*, *72*, *75*–*79*), T cell exhaustion is a state of dysfunction associated with expression of surface inhibitor markers such as PD1, TIM3, TIGIT, and LAG3, with PD1/TIM3 coexpression denoting terminally exhausted T cell populations (*72*, *80*). This state is often characterized by reduced functionality, typically manifested by impaired cytokine production (*69*, *70*, *72*, *75*–*78*). Although exhaustion has been most extensively studied in the context of CD8 T cells, similar gene expression profiles are also seen in exhausted CD4 T cells (*75*). Flow cytometry analysis revealed that 5 days of treatment with DRP-104 significantly reduced the proportion of terminally exhausted PD1^+^ TIM3^+^ CD4 T cells, but not TIM3^+^ PD1^+^ CD8 T cells (Fig. 5E and fig. S5C). Unfortunately, likely due to the blocking effect of anti-PD1 therapy, we were unable to identify PD1^+^ populations in mice treated with anti-PD1 therapy (fig. S5D). Despite not observing changes in CD8^+^ PD1^+^ TIM3^+^ T cells, we did find a reduction in PD1^Int^ TCF7^+^ CD8 T cells in DRP-104–treated animals (Fig. 5F), corresponding to CD8-exhausted progenitor cells (*71*, *81*–*83*). These progenitor cells are thought to give rise to terminally exhausted T cells but can also be rescued to differentiate into memory/effector T cells (*81*, *82*, *84*). It is feasible that the increase in CD8 memory cells observed with DRP-104 treatment is due to driving exhausted progenitor CD8 T cells toward memory cell differentiation. Since T cell exhaustion is induced by prolonged antigen stimulation (*85*) and because of our observation that DRP-104 treatment reduced exhausted progenitor CD8 T cells, we hypothesized that additional days of DRP-104 treatment would drive a difference in the proportion of terminally exhausted CD8 T cells. After 10 days of DRP-104 treatment, when we quantified terminally exhausted CD4 and CD8 populations, we observed that both terminally exhausted CD4 and CD8 populations coexpressing PD1 and TIM3 (Fig. 5, G and H) or coexpressing PD1 and LAG3 (Fig. 5, I and J) were significantly reduced.

A fundamental characteristic of T cell exhaustion is reduced T cell function that is typified by decreased production of cytokines (*70*), such as interferon γ (IFNγ) and tumor necrosis factor α (TNFα), which are key effector molecules in antitumor responses. We have previously demonstrated that these cytokines are suppressed in T cells from *Keap1* mutant tumors compared to WT tumors (*50*). To assess whether DRP-104 treatment improves the function of both CD4 and CD8 T cells, we isolated T cells from *Keap1* mutant tumors treated with DRP-104 and/or anti-PD1 and evaluated their cytokine production after phorbol 12-myristate 13-acetate (PMA)/ionomycin stimulation. Flow cytometry analysis revealed that CD4 and CD8 T cells from DRP-104–treated mice had augmented IFNγ and TNFα production (Fig. 5, K to M), suggesting that the effector function of these T lymphocytes was greatly enhanced by the drug. Specifically, we find that DRP-104 treatment resulted in increased IFNγ^+^ TNFα^+^ double-positive T cells (Fig. 5, L and M). Anti-PD1 alone or the combination of DRP-104 with anti-PD1 did not necessarily increase the expression of these effector cytokines when compared to DRP-104 monotherapy (Fig. 5, L and M). In summary, using complementary methods, including ExCITE-seq and functional flow cytometry assays, we established that DRP-104 not only targets tumor-intrinsic purine metabolism but also diminishes T_regs_ and exhausted T cell populations that characterize *Keap1* mutant lung tumors. On the basis of these observations, we conclude that DRP-104 therapy enhances the functionality of antitumor CD4 and CD8 T cell responses, resulting in overall improved outcomes when combined with anti-PD1 therapy (Fig. 5N).

## DISCUSSION

*KEAP1* mutations are frequently found in LUAD and are associated with poor response rates to standard of care therapy (*2*, *12*, *19*, *65*, *74*). Despite knowing the metabolic vulnerabilities of *KEAP1* mutant LUAD (*2*, *23*, *36*), there are currently no clinically approved treatments specifically targeting this mutation. The KEAPSAKE trial was a phase 2 randomized multicenter double-blind clinical trial comparing the addition of the glutaminase inhibitor CB-839 or placebo control to standard of care checkpoint blockade and chemotherapy for patients with metastatic NSCLC with *KEAP1* or *NRF2* mutations. The trial was terminated due to lack of clinical benefit, and therefore, alternative approaches to targeting *KEAP1* mutant lung tumors need to be explored. Here, using immunodeficient and immunocompetent orthotopic murine cancer models, we demonstrate that DRP-104, a broad-acting glutamine antagonist, is efficacious against *KEAP1* mutant tumors by a mechanism distinct from GLS1-selective inhibitors, such as CB-839. Through our metabolomic analysis, we show that the intrinsic vulnerability of *KEAP1* mutant tumors to DRP-104 arises from the inhibition of nucleotide synthesis. Using our recently developed antigenic orthotopic lung cancer model (*50*), we revealed that DRP-104 not only is effective in targeting *Keap1* mutant lung tumors but also, in combination with checkpoint blockade DRP-104, led to significantly enhanced survival of mice compared to monotherapy. Using ExCITE-seq, we further identified that DRP-104 treatment leads to notable reduction in T_regs_. In addition, combining DRP-104 with anti-PD1 reduces a previously described *Ccl5*-expressing dysfunctional CD8 population (CD8_Ex1_) (*45*) while also expanding memory T cell populations (CD8_Mem_ and CD4_Mem1_). We then validated that DRP-104 reduced exhausted T cell populations and improved the functionality of T cells in the tumor microenvironment, as demonstrated by increased IFNγ and TNFα expression. Overall, our work validates that *Keap1* mutant tumors are sensitive to inhibition of glutamine metabolism by DRP-104, which operates both through cell-intrinsic mechanisms and through enhancement of antitumor T cell responses.

We identified that the major cell-intrinsic mechanism that contributes to the high sensitivity of *KEAP1* mutant tumors to DRP-104 centers on nucleotide synthesis. Previous studies from our laboratory demonstrated that *KEAP1* mutant tumors are glutamine addicted and depend on exogenous glutamine (*2*, *36*). However, unlike CB-839 that impairs glutamate-dependent anaplerosis, DRP-104 targets multiple glutamine-dependent reactions in *KEAP1* mutant tumors. Through a comprehensive metabolic analysis, we identified purine synthesis as a major target of DRP-104. Moreover, supplementation with nucleosides proved sufficient to rescue the DRP-104–mediated inhibition of *Keap1* mutant cell growth. A second possible tumor-intrinsic mechanism contributing to the sensitivity to DRP-104 is due to the enzymatic activation of the prodrug by the enzyme CES1 to produce the active form DON. *CES1* and the mouse ortholog *ces1g* are transcriptional targets of NRF2 (*2*, *47*). As a result, we speculate that the tumor microenvironment of *KEAP1* mutant tumors could be enriched with DON, the active form of DRP-104. This could potentially lead to a significantly enhanced efficacy of the drug. Additional work is needed to validate the role of NRF2 in *Ces1g* expression and DRP-104 sensitivity. CES1 expression could potentially serve as a biomarker in any tumor type that is sensitive to DRP-104. A third potential mechanism of *KEAP1* mutant sensitivity to DRP-104 is through NRF2-mediated regulation of glutamine transporters. NRF2 is known to up-regulate a series of transporters that facilitate glutamine uptake including SLC7A5, SLC38A6, and SLC3A2 (*2*, *86*–*89*). Since DRP-104 and DON are structurally similar to glutamine, the uptake of these drugs into *KEAP1* mutant tumors may be enhanced through increased expression of these glutamine transporters. Down-regulation of these transporters may also reflect a potential mechanism of resistance to DRP-104. Further work investigating the role of NRF2-regulated glutamine transporters can be performed through selective knockout of these transporters.

Targeting glutamine metabolism is a double-edged sword with potential consequences to proliferating cells. Previous work has demonstrated that T cell activation and proliferation is dependent on glutamine (*62*). However, glutamine utilization has multiple effects on T cell effector function (*48*, *61*, *62*, *90*, *91*). It has been suggested that inhibition of GLS1, the enzyme that metabolizes glutamine to glutamate, with CB-839 can either enhance or impair CD8 cytotoxic function (*61*, *90*). Other work has demonstrated that GLS1 inhibition can enhance T_H_1 cytokine production (*49*). The work presented here does not delineate the mechanism in which DRP-104 may reduce T cell exhaustion or enhance T cell function. One hypothesis, referred to as “glutamine steal” phenomenon (*91*), is that tumors with glutamine consumption can inhibit T cell function through depletion of free glutamine in the microenvironment. Potentially, DRP-104 reverses this depletion by inhibiting glutamine consumption of *KEAP1* mutant tumors and thereby increasing extracellular glutamine availability for metabolically active effector T cells. While this hypothesis supports an indirect effect of DRP-104 on T cells, DRP-104 may also have a direct effect on T cells. Previous work has evaluated the effect of DON on T cells (*48*), but did not explore the effect of the prodrug DRP-104. It is also not clear to what extent CES1 is excreted and able to enzymatically activate DRP-104 in the extracellular space of the tumor microenvironment, where it can directly affect T cell function. Further work is needed to investigate the impact of DRP-104 on T cells in a reductionist manner, to specifically examine the impact on T cell activation, cytokine production, and exhaustion.

In summary, DRP-104 is a promising therapeutic agent that has high efficacy in *KEAP1* mutant lung tumors. Our work demonstrates that DRP-104 not only targets tumor-intrinsic vulnerabilities via inhibition of nucleotide synthesis but also enhances the function of antitumor T cells and can be combined with checkpoint blockade, the current standard of care in NSCLC. These findings provide a mechanistic rationale for the clinical trial (NCT04471415) using DRP-104 in combination with checkpoint blockade in LUAD patients specifically with loss-of-function *KEAP1* mutations or gain-of-function *NRF2* mutations.

## MATERIALS AND METHODS

### Cell lines

KP and KPK cells used here were previously established (*2*). *Stk11* knockout tumors were generated by transient transfection of PX458 (Addgene 48138) expressing a guide targeting *Lkb1*. Single green fluorescent protein (GFP)–positive clones were selected, and *Lkb1* loss was validated by Western blot. *Nrf2* gain of function (Neh2 deletion), *Keap1* R470C mutant, and *Keap1* WT KP cell lines were generated as previously described (*2*, *74*). Cells were cultured in Dulbecco’s modified Eagle’s medium (DMEM) with 10% fetal bovine serum (FBS) and gentamicin. *Keap1* R470C and *Keap1* WT KP cells were maintained in hygromycin selection (800 μg/ml).

### In vitro DRP-104 treatments and metabolic rescues

For cell viability assays, cells were plated in a white, opaque 96-well plate with clear bottom at a density of 1000 cells per well in RPMI 1640 with 10% FBS. After attachment, DRP-104, CB-839, or DON was added at the indicated concentrations. After 5 days, cell viability was assessed by CellTiter-Glo. For metabolic rescue experiments, 2000 cells per well were plated in a 12-well plate in RPMI 1640 with 10% FBS. Cells were pretreated with the indicated metabolites for 24 hours and then treated with DRP-104 for 5 days. Cells were stained with a 0.5% crystal violet solution in 20% methanol. Plates were then washed and dried, and crystal violet was eluted in 400 ml of 10% acetic acid. Data are plotted as relative cell growth to vehicle-treated control.

### Tumor mouse models

All experiments were approved by the New York University (NYU) Institutional Animal Care and Use Committee (IA16-01627). In vivo experiments using KP, KPK, KP *Nrf2* GoF, *Stk11* mutant, and *Keap1/Stk11* co-mutant KP cells were performed using nude (JAX strain #002019), NOD SCID Gamma (NSG; JAX strain #005557), of C57BL/6J (JAX strain #000664) mice. Cells [100,000 cells in 100 μl of phosphate-buffered saline (PBS)] were injected subcutaneously into each flank of the mouse. Tumors were measured by calipers, and volume was calculated based on 0.5 × length × width^2^. Once tumor volume was ~100 mm^3^, treatment was initiated. For PDXs, tumors were implanted in the flank of NSG mice as previously described (*2*). To generate orthotopic lung tumors, *Keap1* WT or *Keap1* R470C KP cells expressing luciferase GFP were injected intravenously (100,000 cells in 100 μl of PBS) into female C57BL/6J (JAX strain #000664) mice and tumor burden was measured by bioluminescence (PerkinElmer IVIS Spectrum In Vivo Imaging System, D-luciferin, PerkinElmer #122799). Data were analyzed using Living Image software.

### Treatments and T cell depletion

Treatment with DRP-104 (1 to 4 mg/kg) or vehicle control (10% Tween 80, 10% ethanol in 0.9% saline) was administered subcutaneously 5 days on, 2 days off. For anti-PD1 therapy, mice were administered anti-PD1 (29.F1A12, Bio X Cell BE0273) or isotype control [rat immunoglobulin G2a (IgG2a), Bio X Cell #BE0089] (200 μg) intraperitoneally three times a week. For T cell depletion experiments, either anti-CD8 (2.43, Bio X Cell BE0061) or isotype control (rat IgG2a, LTF-2, Bio X Cell BE0090) (150 μg) was administered intraperitoneally twice a week once tumor burden was established by bioluminescence. One day after administration of antibody, DRP-104 (3 mg/kg) was injected subcutaneously 5 days on, 2 days off.

### Metabolomics

For in vitro tracing, KPK tumor cells were plated at 100,000 cells per well in a 12-well plate in RPMI 1640 with 10% FBS. Cells were treated with DRP-104 [0.5 to 1.0 μM or dimethyl sulfoxide (DMSO)] for 24 hours. The medium was then replaced with fresh RPMI containing either 11 mM [U^13^C]-d-glucose or 2 mM [U^13^C]-l-glutamine and cultured for 1 hour. Cells were collected and prepared for LCMS as previously described (*92*). For in vivo metabolomics, CTG743 PDX tumors were implanted into NSG mice as described above. After tumors were approximately 100 mm^3^, mice were treated with either vehicle control or DRP-104 (3 mg/kg) for 5 days. Mice were euthanized, and then tumors were dissected. Tumor tissue was immediately flash-frozen in liquid nitrogen. Approximately 5 mg of tissue was collected for analysis by LCMS. Tumor tissue was homogenized in metabolite extraction buffer [80% (v/v) ice-cold methanol containing norvaline (1.4 μg/ml)] using Precellys. After homogenization, tissue samples were prepared for LCMS following the same methods used for the in vitro tracing experiments described above. Fractional enrichment was calculated as the peak area of an individual isotopologue divided by the summed peak areas of all isotopologues for that metabolite.

### ExCITE sequencing

Mice were sedated with ketamine and xylazine and then were injected with 2 μg of allophycocyanin (APC) anti-CD45 (2 μg per mouse diluted in 100 μl of PBS, BioLegend 30-F11) retro-orbitally. After 3 min, the chest of the mouse was opened. Lungs were removed and each lobe was separated and cleaned. The lung lobes were cut on a glass slide into small pieces and then digested [collagenase IV (Sigma-Aldrich, C5138), deoxyribonuclease I (Sigma-Aldrich, DN25) in RPMI with 10% FBS] for 35 min at 37°C. Digestion was stopped by addition of EDTA (1 mM). Digested cells were then filtered into a single-cell suspension through a 100-μm filter. Red blood cell (RBC) lysis was performed. Cells were then washed and suspended in a staining buffer. Cells were then stained with live dead staining (Zombie UV fixable viability dye, BioLegend #423107) and phycoerythrin (PE)–Cy7 anti-CD45 (see the next section for staining protocol).

Approximately 500,000 lung immune cells from each condition (two mice per condition) were sorted as live^+^ IV-CD45^−^ CD45^+^, and 50,000 tumor cells were sorted as live^+^ CD45^−^ GFP^+^. Sorted samples were multiplexed using cell hashing antibodies (BioLegend) and stained with ADTs (see antibody table). Cells (25,000) from each treatment condition were pooled and loaded into 10X Chromium. Gene expression, together with Hashtag oligo (HTO) libraries, was processed using Cell Ranger (v5.0.0) in multi-mode. Cell-containing droplets were selected using HTODemux function available in Seurat program. Unique molecular identifier (UMI) count matrices from each modality were imported into the same Seurat object as separate assays. Viable cells were filtered based on having more than 200 genes detected and less than 10% of total UMIs stemming from mitochondrial transcripts. HTO counts were normalized using centered log ratio transformation before hashed samples were demultiplexed using the Seurat::HTODemux function. Protein counts were normalized using centered log ratio transformation. RNA counts were normalized using Seurat::SCTransform function with regressions of cell cycle score, ribosomal, and mitochondrial percentages. Multimodal integration was performed using the weighted-nearest neighbor (WNN) method in Seurat. Briefly, a WNN network was constructed based on modality weights estimated for each cell using Seurat::FindMultiModalNeighbors function with top 40 and top 30 PCs from normalized RNA and protein counts, respectively. A shared nearest neighbor graph was then built based on the first 40 principal components (PCs) followed by identification of cell clusters using Leiden algorithm and Seurat::FindClusters function at multiple resolutions to identify potential rare cell types. Cell types were annotated based on canonical cell type markers and differential expressed genes of each cluster identified using Seurat::FindAllMarkers function with a logistic regression model. Clusters expressing markers of the same cell type were merged into a single cluster. Cell were then projected onto a uniform manifold (*93*) using the top 40 PCs for visualization.

### Flow cytometry and immunohistochemistry

Mice were euthanized, and lungs were digested into a single-cell suspension as described above. Single cells were transferred to a 96-well round bottom plate and resuspended in FACS (fluorescence-activated cell sorting) buffer [0.5% bovine serum albumin (BSA), 0.1% sodium azide, and 1 mM EDTA]. Live/dead staining was initially performed per protocol (Zombie UV fixable viability dye, BioLegend 423107). Cells were then blocked with Fc block (2.4G2, Bio X Cell) for 10 min on ice. Antibody cocktail for surface staining was then added for 15 min on ice, and then samples were washed with FACS buffer. Cells needing intracellular staining for FoxP3 were fixed and permeabilized using the FoxP3 Staining buffer kit (eBioscience 00552300). Intracellular Fc blocking was applied for 10 min on ice and then intracellularly stained with FoxP3 antibody for 1 hour on ice. Cells were then washed and resuspended in FACS buffer. For cytokine staining, single-cell suspension cells were plated on a 96-well flat bottom plate. Cells were stimulated with PMA (0.1 μg/ml, Sigma-Aldrich P-8139), ionomycin (1 μg/ml, Sigma-Aldrich I-0634), GolgiPlug (BD Biosciences 55029, 1:1000), and GolgiStop (BD Biosciences 555029, 1:1000) for 4.5 hours in RPMI with 10% FBS at 37°C. Next, cells were washed, transferred to a 96-well round bottom plate, and resuspended in FACS buffer. Surface staining was done as described above. Cells were then fixed with 2% paraformaldehyde (diluted in FACS buffer) and then permeabilized by 0.5% saponin (diluted in FACS buffer). Cells were blocked intracellularly with Fc block and then stained for 1 hour with cytokine antibody cocktail. Next, the cells were washed and resuspended in FACS buffer. The samples were filtered with a 100-μm filter and then run on BD LSRFortessa. Data were analyzed using FlowJo version 10.

For immunohistochemistry, tissues were fixed in 10% zinc formalin for 48 hours and processed through graded ethanol and xylene and into paraffin in a Leica Peloris automated processor. The iterative multiplex immunostaining protocol was performed on the Leica BondRX automated stainer, according to the manufacturers’ instructions with the antibodies. Briefly, all slides underwent sequential heat retrieval with either Leica Biosystems epitope retrieval 1 solution (ER1, pH 6.0, AR9961) or retrieval 2 solution (ER2, pH 9.0, AR9640), followed by primary and secondary antibody incubations and tyramide signal amplification (TSA) with Opal fluorophores. Primary and secondary antibodies were removed during heat retrieval steps, while fluorophores remained covalently attached to the epitope. Semi-automated image acquisition was performed on a Vectra Polaris multispectral imaging system at 20×. Whole slide unmixed scans were viewed with Akoya Phenochart. Slides were analyzed using QuPath 0.2.3. Antibodies used are listed in table S1.

### Statistics

Statistical analysis was performed using GraphPad Prism v9. All data are expressed as mean plus SEM. Data were analyzed by statistical test indicated in figure legends. All tests were two tailed.

## References

[1] D. Hanahan, R. A. Weinberg, Hallmarks of cancer: The next generation. Cell 144, 646–674 (2011). 21376230 10.1016/j.cell.2011.02.013

[2] R. Romero, V. I. Sayin, S. M. Davidson, M. R. Bauer, S. X. Singh, S. E. LeBoeuf, T. R. Karakousi, D. C. Ellis, A. Bhutkar, F. J. Sánchez-Rivera, L. Subbaraj, B. Martinez, R. T. Bronson, J. R. Prigge, E. E. Schmidt, C. J. Thomas, C. Goparaju, A. Davies, I. Dolgalev, A. Heguy, V. Allaj, J. T. Poirier, A. L. Moreira, C. M. Rudin, H. I. Pass, M. G. Vander Heiden, T. Jacks, T. Papagiannakopoulos, Keap1 loss promotes Kras-driven lung cancer and results in dependence on glutaminolysis. Nat. Med. 23, 1362–1368 (2017). 28967920 10.1038/nm.4407 PMC5677540

[3] L. Lignitto, S. E. LeBoeuf, H. Homer, S. Jiang, M. Askenazi, T. R. Karakousi, H. I. Pass, A. J. Bhutkar, A. Tsirigos, B. Ueberheide, V. I. Sayin, T. Papagiannakopoulos, M. Pagano, Nrf2 activation promotes lung cancer metastasis by inhibiting the degradation of bach1. Cell 178, 316–329.e18 (2019). 31257023 10.1016/j.cell.2019.06.003 PMC6625921

[4] D. B. Shackelford, E. Abt, L. Gerken, D. S. Vasquez, A. Seki, M. Leblanc, L. Wei, M. C. Fishbein, J. Czernin, P. S. Mischel, R. J. Shaw, LKB1 Inactivation dictates therapeutic response of non-small cell lung cancer to the metabolism drug phenformin. Cancer Cell 23, 143–158 (2013). 23352126 10.1016/j.ccr.2012.12.008 PMC3579627

[5] A. T. Shaw, D. W. Kim, K. Nakagawa, T. Seto, L. Crinó, M. J. Ahn, T. de Pas, B. Besse, B. J. Solomon, F. Blackhall, Y. L. Wu, M. Thomas, K. J. O–Byrne, D. Moro-Sibilot, D. R. Camidge, T. Mok, V. Hirsh, G. J. Riely, S. Iyer, V. Tassell, A. Polli, K. D. Wilner, P. A. Jänne, Crizotinib versus chemotherapy in advanced ALK-positive lung cancer. N. Engl. J. Med. 368, 2385–2394 (2013). 23724913 10.1056/NEJMoa1214886

[6] B. J. Solomon, T. Mok, D. W. Kim, Y. L. Wu, K. Nakagawa, T. Mekhail, E. Felip, F. Cappuzzo, J. Paolini, T. Usari, S. Iyer, A. Reisman, K. D. Wilner, J. Tursi, F. Blackhall, First-line crizotinib versus chemotherapy in ALK-positive lung cancer. N. Engl. J. Med. 371, 2167–2177 (2014). 25470694 10.1056/NEJMoa1408440

[7] A. T. Shaw, T. M. Bauer, F. de Marinis, E. Felip, Y. Goto, G. Liu, J. Mazieres, D. W. Kim, T. Mok, A. Polli, H. Thurm, A. M. Calella, G. Peltz, B. J. Solomon, First-line lorlatinib or crizotinib in advanced ALK-positive lung cancer. N. Engl. J. Med. 383, 2018–2029 (2020). 33207094 10.1056/NEJMoa2027187

[8] M. Maemondo, A. Inoue, K. Kobayashi, S. Sugawara, S. Oizumi, H. Isobe, A. Gemma, M. Harada, H. Yoshizawa, I. Kinoshita, Y. Fujita, S. Okinaga, H. Hirano, K. Yoshimori, T. Harada, T. Ogura, M. Ando, H. Miyazawa, T. Tanaka, Y. Saijo, K. Hagiwara, S. Morita, T. Nukiwa, Gefitinib or chemotherapy for non–small-cell lung cancer with mutated EGFR. N. Engl. J. Med. 362, 2380–2388 (2010). 20573926 10.1056/NEJMoa0909530

[9] S. S. Ramalingam, J. Vansteenkiste, D. Planchard, B. C. Cho, J. E. Gray, Y. Ohe, C. Zhou, T. Reungwetwattana, Y. Cheng, B. Chewaskulyong, R. Shah, M. Cobo, K. H. Lee, P. Cheema, M. Tiseo, T. John, M.-C. Lin, F. Imamura, T. Kurata, A. Todd, R. Hodge, M. Saggese, Y. Rukazenkov, J.-C. Soria; FLAURA Investigators, Overall survival with osimertinib in untreated *EGFR*-mutated advanced NSCLC. N. Engl. J. Med. 382, 41–50 (2020). 31751012 10.1056/NEJMoa1913662

[10] F. Skoulidis, B. T. Li, G. K. Dy, T. J. Price, G. S. Falchook, J. Wolf, A. Italiano, M. Schuler, H. Borghaei, F. Barlesi, T. Kato, A. Curioni-Fontecedro, A. Sacher, A. Spira, S. S. Ramalingam, T. Takahashi, B. Besse, A. Anderson, A. Ang, Q. Tran, O. Mather, H. Henary, G. Ngarmchamnanrith, G. Friberg, V. Velcheti, R. Govindan, Sotorasib for lung cancers with KRAS p.G12C mutation. N. Engl. J. Med. 384, 2371–2381 (2021). 34096690 10.1056/NEJMoa2103695 PMC9116274

[11] R. Pillai, M. Hayashi, A.-M. Zavitsanou, T. Papagiannakopoulos, NRF2: KEAPing tumors protected. Cancer Discov. 12, 625–643 (2022). 35101864 10.1158/2159-8290.CD-21-0922 PMC8904278

[12] W. L. Wu, T. Papagiannakopoulos, The pleiotropic role of the KEAP1/NRF2 pathway in cancer. N. Engl. J. Med. 4, 413–435 (2020).

[13] K. Itoh, T. Chiba, S. Takahashi, T. Ishii, K. Igarashi, Y. Katoh, T. Oyake, N. Hayashi, K. Satoh, I. Hatayama, M. Yamamoto, Y. I. Nabeshima, An Nrf2/small Maf heterodimer mediates the induction of phase II detoxifying enzyme genes through antioxidant response elements. Biochem. Biophys. Res. Commun. 236, 313–322 (1997). 9240432 10.1006/bbrc.1997.6943

[14] K. Itoh, N. Wakabayashi, Y. Katoh, T. Ishii, K. Igarashi, J. D. Engel, M. Yamamoto, Keap1 represses nuclear activation of antioxidant responsive elements by Nrf2 through binding to the amino-terminal Neh2 domain. Genes Dev. 13, 76–86 (1999). 9887101 10.1101/gad.13.1.76 PMC316370

[15] M. McMahon, K. Itoh, M. Yamamoto, S. A. Chanas, C. J. Henderson, L. McLellan, C. R. Wolf, C. Cavin, J. D. Hayes, The Cap–n–Collar basic leucine zipper transcription factor Nrf2 (NF-E2 p45-related Factor 2) controls both constitutive and inducible expression of intestinal detoxification and glutathione biosynthetic enzymes. Cancer Res. 61, 3299–3307 (2001). 11309284

[16] N. Wakabayashi, A. T. Dinkova-Kostova, W. D. Holtzclaw, M. I. Kang, A. Kobayashi, M. Yamamoto, T. W. Kensler, P. Talalay, Protection against electrophile and oxidant stress by induction of the phase 2 response: Fate of cysteines of the Keap1 sensor modified by inducers. Proc. Natl. Acad. Sci. U.S.A. 101, 2040–2045 (2004). 14764894 10.1073/pnas.0307301101 PMC357048

[17] A. Kobayashi, M. I. Kang, H. Okawa, M. Ohtsuji, Y. Zenke, T. Chiba, K. Igarashi, M. Yamamoto, Oxidative stress sensor Keap1 functions as an adaptor for Cul3-based E3 ligase to regulate proteasomal degradation of Nrf2. Mol. Cell. Biol. 24, 7130–7139 (2004). 15282312 10.1128/MCB.24.16.7130-7139.2004 PMC479737

[18] F. Skoulidis, L. A. Byers, L. Diao, V. A. Papadimitrakopoulou, P. Tong, J. Izzo, C. Behrens, H. Kadara, E. R. Parra, J. R. Canales, J. Zhang, U. Giri, J. Gudikote, M. A. Cortez, C. Yang, Y. Fan, M. Peyton, L. Girard, K. R. Coombes, C. Toniatti, T. P. Heffernan, M. Choi, G. M. Frampton, V. Miller, J. N. Weinstein, R. S. Herbst, K. K. Wong, J. Zhang, P. Sharma, G. B. Mills, W. K. Hong, J. D. Minna, J. P. Allison, A. Futreal, J. Wang, I. I. Wistuba, J. V. Heymach, Co-occurring genomic alterations define major subsets of kras-mutant lung adenocarcinoma with distinct biology, immune profiles, and therapeutic vulnerabilities. Cancer Discov. 5, 860–877 (2015). 26069186 10.1158/2159-8290.CD-14-1236 PMC4527963

[19] S. Papillon-Cavanagh, P. Doshi, R. Dobrin, J. Szustakowski, A. M. Walsh, STK11 and KEAP1 mutations as prognostic biomarkers in an observational real-world lung adenocarcinoma cohort. ESMO Open 5, e000706 (2020). 32312757 10.1136/esmoopen-2020-000706 PMC7199918

[20] A. Singh, A. Daemen, D. Nickles, S. M. Jeon, O. Foreman, K. Sudini, F. Gnad, S. Lajoie, N. Gour, W. Mitzner, S. Chatterjee, E. J. Choi, B. Ravishankar, A. Rappaport, N. Patil, M. McCleland, L. Johnson, G. Acquaah-Mensah, E. Gabrielson, S. Biswal, G. Hatzivassiliou, NRF2 Activation promotes aggressive lung cancer and associates with poor clinical outcomes. Clin. Cancer Res. 27, 877–888 (2021). 33077574 10.1158/1078-0432.CCR-20-1985 PMC10867786

[21] M. M. Awad, S. Liu, I. I. Rybkin, K. C. Arbour, J. Dilly, V. W. Zhu, M. L. Johnson, R. S. Heist, T. Patil, G. J. Riely, J. O. Jacobson, X. Yang, N. S. Persky, D. E. Root, K. E. Lowder, H. Feng, S. S. Zhang, K. M. Haigis, Y. P. Hung, L. M. Sholl, B. M. Wolpin, J. Wiese, J. Christiansen, J. Lee, A. B. Schrock, L. P. Lim, K. Garg, M. Li, L. D. Engstrom, L. Waters, J. D. Lawson, P. Olson, P. Lito, S. H. I. Ou, J. G. Christensen, P. A. Jänne, A. J. Aguirre, Acquired resistance to KRASG12C inhibition in cancer. N. Engl. J. Med. 384, 2382–2393 (2021). 34161704 10.1056/NEJMoa2105281 PMC8864540

[22] M. V. Negrao, H. A. Araujo, G. Lamberti, A. J. Cooper, N. S. Akhave, T. Zhou, L. Delasos, J. K. Hicks, M. Aldea, G. Minuti, J. Hines, J. V. Aredo, M. J. Dennis, T. Chakrabarti, S. C. Scott, P. Bironzo, M. Scheffler, P. Christopoulos, A. Stenzinger, J. W. Riess, S. Y. Kim, S. B. Goldberg, M. Li, Q. Wang, Y. Qing, Y. Ni, M. T. Do, R. Lee, B. Ricciuti, J. V. Alessi, J. Wang, B. Resuli, L. Landi, S.-C. Tseng, M. Nishino, S. R. Digumarthy, W. Rinsurongkawong, V. Rinsurongkawong, A. A. Vaporciyan, G. R. Blumenschein, J. Zhang, D. H. Owen, C. M. Blakely, G. Mountzios, C. A. Shu, C. M. Bestvina, M. C. Garassino, K. A. Marrone, J. E. Gray, S. P. Patel, A. L. Cummings, H. A. Wakelee, J. Wolf, G. V. Scagliotti, F. Cappuzzo, F. Barlesi, P. D. Patil, L. Drusbosky, D. L. Gibbons, F. Meric-Bernstam, J. J. Lee, J. V. Heymach, D. S. Hong, R. S. Heist, M. M. Awad, F. Skoulidis, Co-mutations and KRAS G12C inhibitor efficacy in advanced NSCLC. Cancer. Discov. 13, 1556–1571 (2023). 37068173 10.1158/2159-8290.CD-22-1420 PMC11024958

[23] R. Romero, F. J. Sánchez-Rivera, P. M. K. Westcott, K. L. Mercer, A. Bhutkar, A. Muir, T. J. González Robles, S. Lamboy Rodríguez, L. Z. Liao, S. R. Ng, L. Li, C. I. Colón, S. Naranjo, M. C. Beytagh, C. A. Lewis, P. P. Hsu, R. T. Bronson, M. G. Vander Heiden, T. Jacks, Keap1 mutation renders lung adenocarcinomas dependent on Slc33a1. Nat. Cancer 1, 589–602 (2020). 34414377 10.1038/s43018-020-0071-1 PMC8373048

[24] S. A. Best, D. P. de Souza, A. Kersbergen, A. N. Policheni, S. Dayalan, D. Tull, V. Rathi, D. H. Gray, M. E. Ritchie, M. J. McConville, K. D. Sutherland, Synergy between the KEAP1/NRF2 and PI3K pathways drives non-small-cell lung cancer with an altered immune microenvironment. Cell Metab. 27, 935–943.e4 (2018). 29526543 10.1016/j.cmet.2018.02.006

[25] S. A. Best, S. Ding, A. Kersbergen, X. Dong, J. Y. Song, Y. Xie, B. Reljic, K. Li, J. E. Vince, V. Rathi, G. M. Wright, M. E. Ritchie, K. D. Sutherland, Distinct initiating events underpin the immune and metabolic heterogeneity of KRAS-mutant lung adenocarcinoma. Nat. Commun. 10, 4190 (2019). 31519898 10.1038/s41467-019-12164-y PMC6744438

[26] K. Chan, F. Robert, C. Oertlin, D. Kapeller-Libermann, D. Avizonis, J. Gutierrez, A. Handly-Santana, M. Doubrovin, J. Park, C. Schoepfer, B. da Silva, M. Yao, F. Gorton, J. Shi, C. J. Thomas, L. E. Brown, J. A. Porco Jr., M. Pollak, O. Larsson, J. Pelletier, I. I. C. Chio, eIF4A supports an oncogenic translation program in pancreatic ductal adenocarcinoma. Nat. Commun. 10, 5151 (2019). 31723131 10.1038/s41467-019-13086-5 PMC6853918

[27] I. I. C. Chio, S. M. Jafarnejad, M. Ponz-Sarvise, Y. Park, K. Rivera, W. Palm, J. Wilson, V. Sangar, Y. Hao, D. Öhlund, K. Wright, D. Filippini, E. J. Lee, B. da Silva, C. Schoepfer, J. E. Wilkinson, J. M. Buscaglia, G. M. DeNicola, H. Tiriac, M. Hammell, H. C. Crawford, E. E. Schmidt, C. B. Thompson, D. J. Pappin, N. Sonenberg, D. A. Tuveson, NRF2 promotes tumor maintenance by modulating mRNA translation in pancreatic cancer. Cell 166, 963–976 (2016). 27477511 10.1016/j.cell.2016.06.056 PMC5234705

[28] G. M. DeNicola, P. H. Chen, E. Mullarky, J. A. Sudderth, Z. Hu, D. Wu, H. Tang, Y. Xie, J. M. Asara, K. E. Huffman, I. I. Wistuba, J. D. Minna, R. J. DeBerardinis, L. C. Cantley, NRF2 regulates serine biosynthesis in non–small cell lung cancer. Nat. Genet. 47, 1475–1481 (2015). 26482881 10.1038/ng.3421 PMC4721512

[29] G. M. DeNicola, F. A. Karreth, T. J. Humpton, A. Gopinathan, C. Wei, K. Frese, D. Mangal, K. H. Yu, C. J. Yeo, E. S. Calhoun, F. Scrimieri, J. M. Winter, R. H. Hruban, C. Iacobuzio-Donahue, S. E. Kern, I. A. Blair, D. A. Tuveson, Oncogene-induced Nrf2 transcription promotes ROS detoxification and tumorigenesis. Nature 475, 106–109 (2011). 21734707 10.1038/nature10189 PMC3404470

[30] Y. P. Kang, L. Torrente, A. Falzone, C. M. Elkins, M. Liu, J. M. Asara, C. C. Dibble, G. M. DeNicola, Cysteine dioxygenase 1 is a metabolic liability for non-small cell lung cancer. eLife 8, e45572 (2019). 31107239 10.7554/eLife.45572 PMC6584702

[31] A. T. Dinkova-Kostova, W. D. Holtzclaw, R. N. Cole, K. Itoh, N. Wakabayashi, Y. Katoh, M. Yamamoto, P. Talalay, Direct evidence that sulfhydryl groups of Keap1 are the sensors regulating induction of phase 2 enzymes that protect against carcinogens and oxidants. Proc. Natl. Acad. Sci. U.S.A. 99, 11908–11913 (2002). 12193649 10.1073/pnas.172398899 PMC129367

[32] T. Shibata, A. Kokubu, M. Gotoh, H. Ojima, T. Ohta, M. Yamamoto, S. Hirohashi, Genetic alteration of Keap1 confers constitutive Nrf2 activation and resistance to chemotherapy in gallbladder cancer. Gastroenterology 135, 1358–1368.e4 (2008). 18692501 10.1053/j.gastro.2008.06.082

[33] S. Homma, Y. Ishii, Y. Morishima, T. Yamadori, Y. Matsuno, N. Haraguchi, N. Kikuchi, H. Satoh, T. Sakamoto, N. Hizawa, K. Itoh, M. Yamamoto, Nrf2 Enhances cell proliferation and resistance to anticancer drugs in human lung cancer. Clin. Cancer Res. 15, 3423–3432 (2009). 19417020 10.1158/1078-0432.CCR-08-2822

[34] T. Saito, Y. Ichimura, K. Taguchi, T. Suzuki, T. Mizushima, K. Takagi, Y. Hirose, M. Nagahashi, T. Iso, T. Fukutomi, M. Ohishi, K. Endo, T. Uemura, Y. Nishito, S. Okuda, M. Obata, T. Kouno, R. Imamura, Y. Tada, R. Obata, D. Yasuda, K. Takahashi, T. Fujimura, J. Pi, M. S. Lee, T. Ueno, T. Ohe, T. Mashino, T. Wakai, H. Kojima, T. Okabe, T. Nagano, H. Motohashi, S. Waguri, T. Soga, M. Yamamoto, K. Tanaka, M. Komatsu, p62/Sqstm1 promotes malignancy of HCV-positive hepatocellular carcinoma through Nrf2-dependent metabolic reprogramming. Nat. Commun. 7, 12030 (2016). 27345495 10.1038/ncomms12030 PMC4931237

[35] M. Hayashi, A. Kuga, M. Suzuki, H. Panda, H. Kitamura, H. Motohashi, M. Yamamoto, Microenvironmental activation of Nrf2 restricts the progression of Nrf2-activated malignant tumors. Cancer Res. 80, 3331–3344 (2020). 32636316 10.1158/0008-5472.CAN-19-2888

[36] V. I. Sayin, S. E. LeBoeuf, S. X. Singh, S. M. Davidson, D. Biancur, B. S. Guzelhan, S. W. Alvarez, W. L. Wu, T. R. Karakousi, A. M. Zavitsanou, J. Ubriaco, A. Muir, D. Karagiannis, P. J. Morris, C. J. Thomas, R. Possemato, M. G. Vander Heiden, T. Papagiannakopoulos, Activation of the NRF2 antioxidant program generates an imbalance in central carbon metabolism in cancer. eLife 6, e28083 (2017). 28967864 10.7554/eLife.28083 PMC5624783

[37] S. Mukhopadhyay, D. Goswami, P. P. Adiseshaiah, W. Burgan, M. Yi, T. M. Guerin, S. V. Kozlov, D. V. Nissley, F. McCormick, Undermining glutaminolysis bolsters chemotherapy while NRF2 promotes chemoresistance in KRAS-driven pancreatic cancers. Cancer Res. 80, 1630–1643 (2020). 31911550 10.1158/0008-5472.CAN-19-1363 PMC7185043

[38] H. W. Dion, S. A. Fusari, Z. L. Jakubowski, J. G. Zora, Q. R. Bartz, 6-Diazo-5-oxo-L-norleucine, a new tumor-inhibitory substance. II. Isolation and characterization. J. Am. Chem. Soc. 78, 3075–3077 (1956).

[39] A. Rahman, F. P. Smith, P. T. Luc, P. V. Woolley, Phase I study and clinical pharmacology of 6-diazo-5-oxo-L-norleucine (DON). Invest. New Drugs 3, 369–374 (1985). 4086244 10.1007/BF00170760

[40] G. Lynch, N. Kemeny, E. Casper, Phase II evaluation of DON (6-diazo-5-oxo-L-norleucine) in patients with advanced colorectal carcinoma. Am. J. Clin. Oncol. 5, 541–543 (1982). 7180833

[41] G. B. Magill, W. P. L. Myers, H. C. Reilly, R. C. Putnam, J. W. Magill, M. P. Sykes, G. C. Escher, D. A. Karnofsky, J. H. Burchenal, Pharmacological and initial therapeutic observations on 6-diazo-5-oxo-1-norleucine (DON) in human neoplastic disease. Cancer 10, 1138–1150 (1957). 13489662 10.1002/1097-0142(195711/12)10:6<1138::aid-cncr2820100608>3.0.co;2-k

[42] K. M. Lemberg, J. J. Vornov, R. Rais, B. S. Slusher, We–re not “DON” yet: Optimal dosing and prodrug delivery of 6-diazo-5-oxo-L-norleucine. Mol. Cancer Ther. 17, 1824–1832 (2018). 30181331 10.1158/1535-7163.MCT-17-1148 PMC6130910

[43] R. Rais, K. M. Lemberg, L. Tenora, M. L. Arwood, A. Pal, J. Alt, Y. Wu, J. Lam, J. M. H. Aguilar, L. Zhao, D. E. Peters, C. Tallon, R. Pandey, A. G. Thomas, R. P. Dash, T. Seiwert, P. Majer, R. D. Leone, J. D. Powell, B. S. Slusher, Discovery of DRP-104, a tumor-targeted metabolic inhibitor prodrug. Sci. Adv. 8, eabq5925 (2022). 36383674 10.1126/sciadv.abq5925 PMC9668306

[44] Y. Yokoyama, T. M. Estok, R. Wild, Sirpiglenastat (DRP-104) induces antitumor efficacy through direct, broad antagonism of glutamine metabolism and stimulation of the innate and adaptive immune systems. Mol. Cancer Ther. 21, 1561–1572 (2022). 35930753 10.1158/1535-7163.MCT-22-0282

[45] B. L. Horton, D. M. Morgan, N. Momin, M. Zagorulya, E. Torres-Mejia, V. Bhandarkar, K. D. Wittrup, J. C. Love, S. Spranger, Lack of CD8+ T cell effector differentiation during priming mediates checkpoint blockade resistance in non-small cell lung cancer. Sci. Immunol. 6, eabi8800 (2021). 34714687 10.1126/sciimmunol.abi8800 PMC10786005

[46] V. I. Sayin, M. X. Ibrahim, E. Larsson, J. A. Nilsson, P. Lindahl, M. O. Bergo, Antioxidants accelerate lung cancer progression in mice. Sci. Transl. Med. 6, 221ra215 (2014). 10.1126/scitranslmed.3007653 24477002

[47] E. V. Knatko, M. H. Tatham, Y. Zhang, C. Castro, M. Higgins, S. Dayalan Naidu, C. Leonardi, L. de la Vega, T. Honda, J. L. Griffin, R. T. Hay, A. T. Dinkova-Kostova, Downregulation of Keap1 confers features of a fasted metabolic state. iScience 23, 101638 (2020). 33103077 10.1016/j.isci.2020.101638 PMC7575887

[48] R. D. Leone, L. Zhao, J. M. Englert, I. M. Sun, M. H. Oh, I. H. Sun, M. L. Arwood, I. A. Bettencourt, C. H. Patel, J. Wen, A. Tam, R. L. Blosser, E. Prchalova, J. Alt, R. Rais, B. S. Slusher, J. D. Powell, Glutamine blockade induces divergent metabolic programs to overcome tumor immune evasion. Science 366, 1013–1021 (2019). 31699883 10.1126/science.aav2588 PMC7023461

[49] M. O. Johnson, M. M. Wolf, M. Z. Madden, G. Andrejeva, A. Sugiura, D. C. Contreras, D. Maseda, M. V. Liberti, K. Paz, R. J. Kishton, M. E. Johnson, A. A. de Cubas, P. Wu, G. Li, Y. Zhang, D. C. Newcomb, A. D. Wells, N. P. Restifo, W. K. Rathmell, J. W. Locasale, M. L. Davila, B. R. Blazar, J. C. Rathmell, Distinct regulation of Th17 and Th1 cell differentiation by glutaminase-dependent metabolism. Cell 175, 1780–1795.e19 (2018). 30392958 10.1016/j.cell.2018.10.001 PMC6361668

[50] A.-M. Zavitsanou, R. Pillai, Y. Hao, W. L. Wu, E. Bartnicki, T. Karakousi, S. Rajalingam, A. Herrera, A. Karatza, A. Rashidfarrokhi, S. Solis, M. Ciampricotti, A. H. Yeaton, E. Ivanova, C. A. Wohlhieter, T. B. Buus, M. Hayashi, B. Karadal-Ferrena, H. I. Pass, J. T. Poirier, C. M. Rudin, K. K. Wong, A. L. Moreira, K. M. Khanna, A. Tsirigos, T. Papagiannakopoulos, S. B. Koralov, KEAP1 mutation in lung adenocarcinoma promotes immune evasion and immunotherapy resistance. Cell Rep. 42, (2023). 10.1016/j.celrep.2023.113295 PMC10755970 37889752

[51] B. J. Altman, Z. E. Stine, C. V. Dang, From Krebs to clinic: Glutamine metabolism to cancer therapy. Nat. Rev. Cancer 16, 619–634 (2016). 27492215 10.1038/nrc.2016.71 PMC5484415

[52] F. Huang, M. Ni, M. D. Chalishazar, K. E. Huffman, J. Kim, L. Cai, X. Shi, F. Cai, L. G. Zacharias, A. S. Ireland, K. Li, W. Gu, A. K. Kaushik, X. Liu, A. F. Gazdar, T. G. Oliver, J. D. Minna, Z. Hu, R. J. DeBerardinis, Inosine monophosphate dehydrogenase dependence in a subset of small cell lung cancers. Cell Metab. 28, 369–382.e5 (2018). 30043754 10.1016/j.cmet.2018.06.005 PMC6125205

[53] A. K. Kaushik, A. Tarangelo, L. K. Boroughs, M. Ragavan, Y. Zhang, C.-Y. Wu, X. Li, K. Ahumada, J.-C. Chiang, V. T. Tcheuyap, F. Saatchi, Q. N. Do, C. Yong, T. Rosales, C. Stevens, A. D. Rao, B. Faubert, P. Pachnis, L. G. Zacharias, H. Vu, F. Cai, T. P. Mathews, G. Genovese, B. S. Slusher, P. Kapur, X. Sun, M. Merritt, J. Brugarolas, R. J. De Berardinis, In vivo characterization of glutamine metabolism identifies therapeutic targets in clear cell renal cell carcinoma. Sci. Adv. 8, eabp8293 (2022). 36525494 10.1126/sciadv.abp8293 PMC9757752

[54] C.-H. Chang, J. Qiu, D. O’Sullivan, M. D. Buck, T. Noguchi, J. D. Curtis, Q. Chen, M. Gindin, M. M. Gubin, G. J. W. van der Windt, E. Tonc, R. D. Schreiber, E. J. Pearce, E. L. Pearce, Metabolic competition in the tumor microenvironment is a driver of cancer progression. Cell 162, 1229–1241 (2015). 26321679 10.1016/j.cell.2015.08.016 PMC4864363

[55] E. H. Ma, G. Bantug, T. Griss, S. Condotta, R. M. Johnson, B. Samborska, N. Mainolfi, V. Suri, H. Guak, M. L. Balmer, M. J. Verway, T. C. Raissi, H. Tsui, G. Boukhaled, S. Henriques da Costa, C. Frezza, C. M. Krawczyk, A. Friedman, M. Manfredi, M. J. Richer, C. Hess, R. G. Jones, Serine is an essential metabolite for effector T cell expansion. Cell Metab. 25, 345–357 (2017). 28111214 10.1016/j.cmet.2016.12.011

[56] R. D. Sheldon, E. H. Ma, L. M. DeCamp, K. S. Williams, R. G. Jones, Interrogating in vivo T-cell metabolism in mice using stable isotope labeling metabolomics and rapid cell sorting. Nat. Protoc. 16, 4494–4521 (2021). 34349284 10.1038/s41596-021-00586-2

[57] B. Samborska, D. G. Roy, J. F. Rahbani, M. F. Hussain, E. H. Ma, R. G. Jones, L. Kazak, Creatine transport and creatine kinase activity is required for CD8+ T cell immunity. Cell Rep. 38, 110446 (2022). 35235777 10.1016/j.celrep.2022.110446

[58] I. Kaymak, K. M. Luda, L. R. Duimstra, E. H. Ma, J. Longo, M. S. Dahabieh, B. Faubert, B. M. Oswald, M. L. J. Watson, S. M. Kitchen-Goosen, L. M. DeCamp, S. E. Compton, Z. Fu, R. J. DeBerardinis, K. S. Williams, R. D. Sheldon, R. G. Jones, Carbon source availability drives nutrient utilization in CD8+ T cells. Cell Metab. 34, 1298–1311.e6 (2022). 35981545 10.1016/j.cmet.2022.07.012 PMC10068808

[59] C.-H. Chang, J. D. Curtis, L. B. Maggi Jr., B. Faubert, A. V. Villarino, D. O’Sullivan, S. C. C. Huang, G. J. W. van der Windt, J. Blagih, J. Qiu, J. D. Weber, E. J. Pearce, R. G. Jones, E. L. Pearce, Posttranscriptional control of T Cell effector function by aerobic glycolysis. Cell 153, 1239–1251 (2013). 23746840 10.1016/j.cell.2013.05.016 PMC3804311

[60] J. Wu, G. Li, L. Li, D. Li, Z. Dong, P. Jiang, Asparagine enhances LCK signalling to potentiate CD8+ T-cell activation and anti-tumour responses. Nat. Cell Biol. 23, 75–86 (2021). 33420490 10.1038/s41556-020-00615-4

[61] S. A. Best, P. M. Gubser, S. Sethumadhavan, A. Kersbergen, Y. L. Negrón Abril, J. Goldford, K. Sellers, W. Abeysekera, A. L. Garnham, J. A. McDonald, C. E. Weeden, D. Anderson, D. Pirman, T. P. Roddy, D. J. Creek, A. Kallies, G. Kingsbury, K. D. Sutherland, Glutaminase inhibition impairs CD8 T cell activation in STK11-/Lkb1-deficient lung cancer. Cell Metab. 34, 874–887.e6 (2022). 35504291 10.1016/j.cmet.2022.04.003

[62] E. L. Carr, A. Kelman, G. S. Wu, R. Gopaul, E. Senkevitch, A. Aghvanyan, A. M. Turay, K. A. Frauwirth, Glutamine uptake and metabolism are coordinately regulated by ERK/MAPK during T lymphocyte activation. J. Immunol. 185, 1037–1044 (2010). 20554958 10.4049/jimmunol.0903586 PMC2897897

[63] M. Momcilovic, S. T. Bailey, J. T. Lee, M. C. Fishbein, C. Magyar, D. Braas, T. Graeber, N. J. Jackson, J. Czernin, E. Emberley, M. Gross, J. Janes, A. Mackinnon, A. Pan, M. Rodriguez, M. Works, W. Zhang, F. Parlati, S. Demo, E. Garon, K. Krysan, T. C. Walser, S. M. Dubinett, S. Sadeghi, H. R. Christofk, D. B. Shackelford, Targeted inhibition of EGFR and glutaminase induces metabolic crisis in EGFR mutant lung cancer. Cell Rep. 18, 601–610 (2017). 28099841 10.1016/j.celrep.2016.12.061 PMC5260616

[64] M. Momcilovic, S. T. Bailey, J. T. Lee, M. C. Fishbein, D. Braas, J. Go, T. G. Graeber, F. Parlati, S. Demo, R. Li, T. C. Walser, M. Gricowski, R. Shuman, J. Ibarra, D. Fridman, M. E. Phelps, K. Badran, M. St. John, N. M. Bernthal, N. Federman, J. Yanagawa, S. M. Dubinett, S. Sadeghi, H. R. Christofk, D. B. Shackelford, The GSK3 signaling axis regulates adaptive glutamine metabolism in lung squamous cell carcinoma. Cancer Cell 33, 905–921.e5 (2018). 29763624 10.1016/j.ccell.2018.04.002 PMC6451645

[65] D. Marinelli, M. Mazzotta, S. Scalera, I. Terrenato, F. Sperati, L. D’Ambrosio, M. Pallocca, G. Corleone, E. Krasniqi, L. Pizzuti, M. Barba, S. Carpano, P. Vici, M. Filetti, R. Giusti, A. Vecchione, M. Occhipinti, A. Gelibter, A. Botticelli, F. De Nicola, L. Ciuffreda, F. Goeman, E. Gallo, P. Visca, E. Pescarmona, M. Fanciulli, R. De Maria, P. Marchetti, G. Ciliberto, M. Maugeri-Saccà, KEAP1-driven co-mutations in lung adenocarcinoma unresponsive to immunotherapy despite high tumor mutational burden. Ann. Oncol. 31, 1746–1754 (2020). 32866624 10.1016/j.annonc.2020.08.2105

[66] E. P. Mimitou, A. Cheng, A. Montalbano, S. Hao, M. Stoeckius, M. Legut, T. Roush, A. Herrera, E. Papalexi, Z. Ouyang, R. Satija, N. E. Sanjana, S. B. Koralov, P. Smibert, Multiplexed detection of proteins, transcriptomes, clonotypes and CRISPR perturbations in single cells. Nat. Methods 16, 409–412 (2019). 31011186 10.1038/s41592-019-0392-0 PMC6557128

[67] T. B. Buus, A. Herrera, E. Ivanova, E. Mimitou, A. Cheng, R. S. Herati, T. Papagiannakopoulos, P. Smibert, N. Odum, S. B. Koralov, Improving oligo-conjugated antibody signal in multimodal single-cell analysis. eLife 10, e61973 (2021). 33861199 10.7554/eLife.61973 PMC8051954

[68] M. Stoeckius, C. Hafemeister, W. Stephenson, B. Houck-Loomis, P. K. Chattopadhyay, H. Swerdlow, R. Satija, P. Smibert, Simultaneous epitope and transcriptome measurement in single cells. Nat. Methods 14, 865–868 (2017). 28759029 10.1038/nmeth.4380 PMC5669064

[69] A. Schietinger, M. Philip, V. E. Krisnawan, E. Y. Chiu, J. J. Delrow, R. S. Basom, P. Lauer, D. G. Brockstedt, S. E. Knoblaugh, G. J. Hämmerling, T. D. Schell, N. Garbi, P. D. Greenberg, Tumor-specific T cell dysfunction is a dynamic antigen-driven differentiation program initiated early during tumorigenesis. Immunity 45, 389–401 (2016). 27521269 10.1016/j.immuni.2016.07.011 PMC5119632

[70] E. J. Wherry, S. J. Ha, S. M. Kaech, W. N. Haining, S. Sarkar, V. Kalia, S. Subramaniam, J. N. Blattman, D. L. Barber, R. Ahmed, Molecular signature of CD8+ T cell exhaustion during chronic viral infection. Immunity 27, 670–684 (2007). 17950003 10.1016/j.immuni.2007.09.006

[71] M. A. Paley, D. C. Kroy, P. M. Odorizzi, J. B. Johnnidis, D. V. Dolfi, B. E. Barnett, E. K. Bikoff, E. J. Robertson, G. M. Lauer, S. L. Reiner, E. J. Wherry, Progenitor and terminal subsets of CD8+ T cells cooperate to contain chronic viral infection. Science 338, 1220–1225 (2012). 23197535 10.1126/science.1229620 PMC3653769

[72] L. M. McLane, M. S. Abdel-Hakeem, E. J. Wherry, CD8 T Cell exhaustion during chronic viral infection and cancer. Annu. Rev. Immunol. 37, 457–495 (2015). 10.1146/annurev-immunol-041015-055318 30676822

[73] T. L. Walunas, D. S. Bruce, L. Dustin, D. Y. Loh, J. A. Bluestone, Ly-6C is a marker of memory CD8+ T cells. J. Immunol. 155, 1873–1883 (1995). 7543536

[74] A.-M. Zavitsanou, R. Pillai, Y. Hao, W. L. Wu, E. Bartnicki, T. Karakousi, S. Rajalingam, A. Herrera, A. Karatza, A. Rashidfarrokhi, S. Solis, M. Ciampricotti, A. H. Yeaton, E. Ivanova, C. A. Wohlhieter, T. B. Buus, M. Hayashi, B. Karadal-Ferrena, H. I. Pass, J. T. Poirier, C. M. Rudin, K.-K. Wong, A. L. Moreira, K. M. Khanna, A. Tsirigos, T. Papagiannakopoulos, S. B. Koralov, KEAP1 mutation in lung adenocarcinoma promotes immune evasion and immunotherapy resistance. Cell Rep. 42, (2023). 10.1016/j.celrep.2023.113295 PMC10755970 37889752

[75] A. Crawford, J. M. Angelosanto, C. Kao, T. A. Doering, P. M. Odorizzi, B. E. Barnett, E. J. Wherry, Molecular and transcriptional basis of CD4+ T Cell dysfunction during chronic infection. Immunity 40, 289–302 (2014). 24530057 10.1016/j.immuni.2014.01.005 PMC3990591

[76] M. Philip, L. Fairchild, L. Sun, E. L. Horste, S. Camara, M. Shakiba, A. C. Scott, A. Viale, P. Lauer, T. Merghoub, M. D. Hellmann, J. D. Wolchok, C. S. Leslie, A. Schietinger, Chromatin states define tumour-specific T cell dysfunction and reprogramming. Nature 545, 452–456 (2017). 28514453 10.1038/nature22367 PMC5693219

[77] M. Philip, A. Schietinger, Heterogeneity and fate choice: T cell exhaustion in cancer and chronic infections. Curr. Opin. Immunol. 58, 98–103 (2019). 31181510 10.1016/j.coi.2019.04.014 PMC7608527

[78] A. C. Scott, F. Dündar, P. Zumbo, S. S. Chandran, C. A. Klebanoff, M. Shakiba, P. Trivedi, L. Menocal, H. Appleby, S. Camara, D. Zamarin, T. Walther, A. Snyder, M. R. Femia, E. A. Comen, H. Y. Wen, M. D. Hellmann, N. Anandasabapathy, Y. Liu, N. K. Altorki, P. Lauer, O. Levy, M. S. Glickman, J. Kaye, D. Betel, M. Philip, A. Schietinger, TOX is a critical regulator of tumour-specific T cell differentiation. Nature 571, 270–274 (2019). 31207604 10.1038/s41586-019-1324-y PMC7698992

[79] M. Andreatta, J. Corria-Osorio, S. Müller, R. Cubas, G. Coukos, S. J. Carmona, Interpretation of T cell states from single-cell transcriptomics data using reference atlases. Nat. Commun. 12, 2965 (2021). 34017005 10.1038/s41467-021-23324-4 PMC8137700

[80] C. U. Blank, W. N. Haining, W. Held, P. G. Hogan, A. Kallies, E. Lugli, R. C. Lynn, M. Philip, A. Rao, N. P. Restifo, A. Schietinger, T. N. Schumacher, P. L. Schwartzberg, A. H. Sharpe, D. E. Speiser, E. J. Wherry, B. A. Youngblood, D. Zehn, Defining ‘T cell exhaustion’. Nat. Rev. Immunol. 19, 665–674 (2019). 31570879 10.1038/s41577-019-0221-9 PMC7286441

[81] D. T. Utzschneider, M. Charmoy, V. Chennupati, L. Pousse, D. P. Ferreira, S. Calderon-Copete, M. Danilo, F. Alfei, M. Hofmann, D. Wieland, S. Pradervand, R. Thimme, D. Zehn, W. Held, T cell factor 1-expressing memory-like CD8(+) T cells sustain the immune response to chronic viral infections. Immunity 45, 415–427 (2016). 27533016 10.1016/j.immuni.2016.07.021

[82] S. J. Im, M. Hashimoto, M. Y. Gerner, J. Lee, H. T. Kissick, M. C. Burger, Q. Shan, J. S. Hale, J. Lee, T. H. Nasti, A. H. Sharpe, G. J. Freeman, R. N. Germain, H. I. Nakaya, H. H. Xue, R. Ahmed, Defining CD8+ T cells that provide the proliferative burst after PD-1 therapy. Nature 537, 417–421 (2016). 27501248 10.1038/nature19330 PMC5297183

[83] B. C. Miller, D. R. Sen, R. al Abosy, K. Bi, Y. V. Virkud, M. W. LaFleur, K. B. Yates, A. Lako, K. Felt, G. S. Naik, M. Manos, E. Gjini, J. R. Kuchroo, J. J. Ishizuka, J. L. Collier, G. K. Griffin, S. Maleri, D. E. Comstock, S. A. Weiss, F. D. Brown, A. Panda, M. D. Zimmer, R. T. Manguso, F. S. Hodi, S. J. Rodig, A. H. Sharpe, W. N. Haining, Subsets of exhausted CD8+ T cells differentially mediate tumor control and respond to checkpoint blockade. Nat. Immunol. 20, 326–336 (2019). 30778252 10.1038/s41590-019-0312-6 PMC6673650

[84] I. Siddiqui, K. Schaeuble, V. Chennupati, S. A. Fuertes Marraco, S. Calderon-Copete, D. Pais Ferreira, S. J. Carmona, L. Scarpellino, D. Gfeller, S. Pradervand, S. A. Luther, D. E. Speiser, W. Held, Intratumoral Tcf1+PD-1+CD8+ T Cells with stem-like properties promote tumor control in response to vaccination and checkpoint blockade immunotherapy. Immunity 50, 195–211.e10 (2019). 30635237 10.1016/j.immuni.2018.12.021

[85] S. A. Vardhana, M. A. Hwee, M. Berisa, D. K. Wells, K. E. Yost, B. King, M. Smith, P. S. Herrera, H. Y. Chang, A. T. Satpathy, M. R. M. van den Brink, J. R. Cross, C. B. Thompson, Impaired mitochondrial oxidative phosphorylation limits the self-renewal of T cells exposed to persistent antigen. Nat. Immunol. 21, 1022–1033 (2020). 32661364 10.1038/s41590-020-0725-2 PMC7442749

[86] Y. D. Bhutia, V. Ganapathy, Glutamine transporters in mammalian cells and their functions in physiology and cancer. Biochim. Biophys. Acta, Mol. Cell Res. 1863, 2531–2539 (2016). 10.1016/j.bbamcr.2015.12.017 PMC4919214 26724577

[87] M. Nachef, A. K. Ali, S. M. Almutairi, S.-H. Lee, Targeting SLC1A5 and SLC3A2/SLC7A5 as a potential strategy to strengthen anti-tumor immunity in the tumor microenvironment. Front. Immunol. 12, 624324 (2021). 33953707 10.3389/fimmu.2021.624324 PMC8089370

[88] Y. Peng, W. Chen, F. Huang, M. Geng, X. Li, F. Zhang, W. Zhu, L. Meng, R. Holmdahl, J. Xu, S. Lu, SLC38A6 expression in macrophages exacerbates pulmonary inflammation. Respir. Res. 24, 33 (2023). 36707853 10.1186/s12931-023-02330-8 PMC9881254

[89] X. Zhao, L. Jin, Y. Liu, Z. Liu, Q. Liu, Bioinformatic analysis of the role of solute carrier-glutamine transporters in breast cancer. Ann. Transl. Med. 10, 777 (2022). 35965834 10.21037/atm-22-2620 PMC9372701

[90] S. Varghese, S. Pramanik, L. J. Williams, H. R. Hodges, C. W. Hudgens, G. M. Fischer, C. K. Luo, B. Knighton, L. Tan, P. L. Lorenzi, A. L. Mackinnon, J. L. McQuade, Y. Hailemichael, J. Roszik, W. Peng, Y. N. Vashisht Gopal, The glutaminase inhibitor CB-839 (Telaglenastat) enhances the antimelanoma activity of T-cell–mediated immunotherapies. Mol. Cancer Ther. 20, 500–511 (2021). 33361272 10.1158/1535-7163.MCT-20-0430 PMC7933078

[91] D. N. Edwards, V. M. Ngwa, A. L. Raybuck, S. Wang, Y. Hwang, L. C. Kim, S. H. Cho, Y. Paik, Q. Wang, S. Zhang, H. C. Manning, J. C. Rathmell, R. S. Cook, M. R. Boothby, J. Chen, Selective glutamine metabolism inhibition in tumor cells improves antitumor T lymphocyte activity in triple-negative breast cancer. J. Clin. Invest. 131, (2021). 10.1172/JCI140100 PMC7880417 33320840

[92] S. E. LeBoeuf, W. L. Wu, T. R. Karakousi, B. Karadal, S. R. E. Jackson, S. M. Davidson, K. K. Wong, S. B. Koralov, V. I. Sayin, T. Papagiannakopoulos, Activation of oxidative stress response in cancer generates a druggable dependency on exogenous non-essential amino acids. Cell Metab. 31, 339–350.e4 (2020). 31813821 10.1016/j.cmet.2019.11.012 PMC7004873

[93] L. H. McInnes, L. M. Innes, J. Healy, N. Saul, L. Großberger, UMAP: Uniform manifold approximation and projection. J. Open Source Softw. 3, 861 (2018).

