# Supplementary material for: Glutamine antagonist DRP-104 suppresses tumor growth and enhances response to checkpoint blockade in KEAP1 mutant lung cancer
Source: Sci Adv. 2024 Mar 27;10(13):eadm9859. doi: 10.1126/sciadv.adm9859 (PMC10971495; doi:10.1126/sciadv.adm9859)
Supplement: Supplementary file 1 — Figs. S1 to S5 Table S1 [file sciadv.adm9859_sm.pdf]

Science Advances
AAAS

Supplementary Materials for

Glutamine antagonist DRP-104 suppresses tumor growth and enhances response to checkpoint blockade in *KEAP1* mutant lung cancer

Ray Pillai *et al.*

Corresponding author: Thales Papagiannakopoulos, thales.papagiannakopoulos@nyulangone.org

Sci. Adv. **10**, eadm9859 (2024)
DOI: 10.1126/sciadv.adm9859

This PDF file includes:

Figs. S1 to S5
Table S1

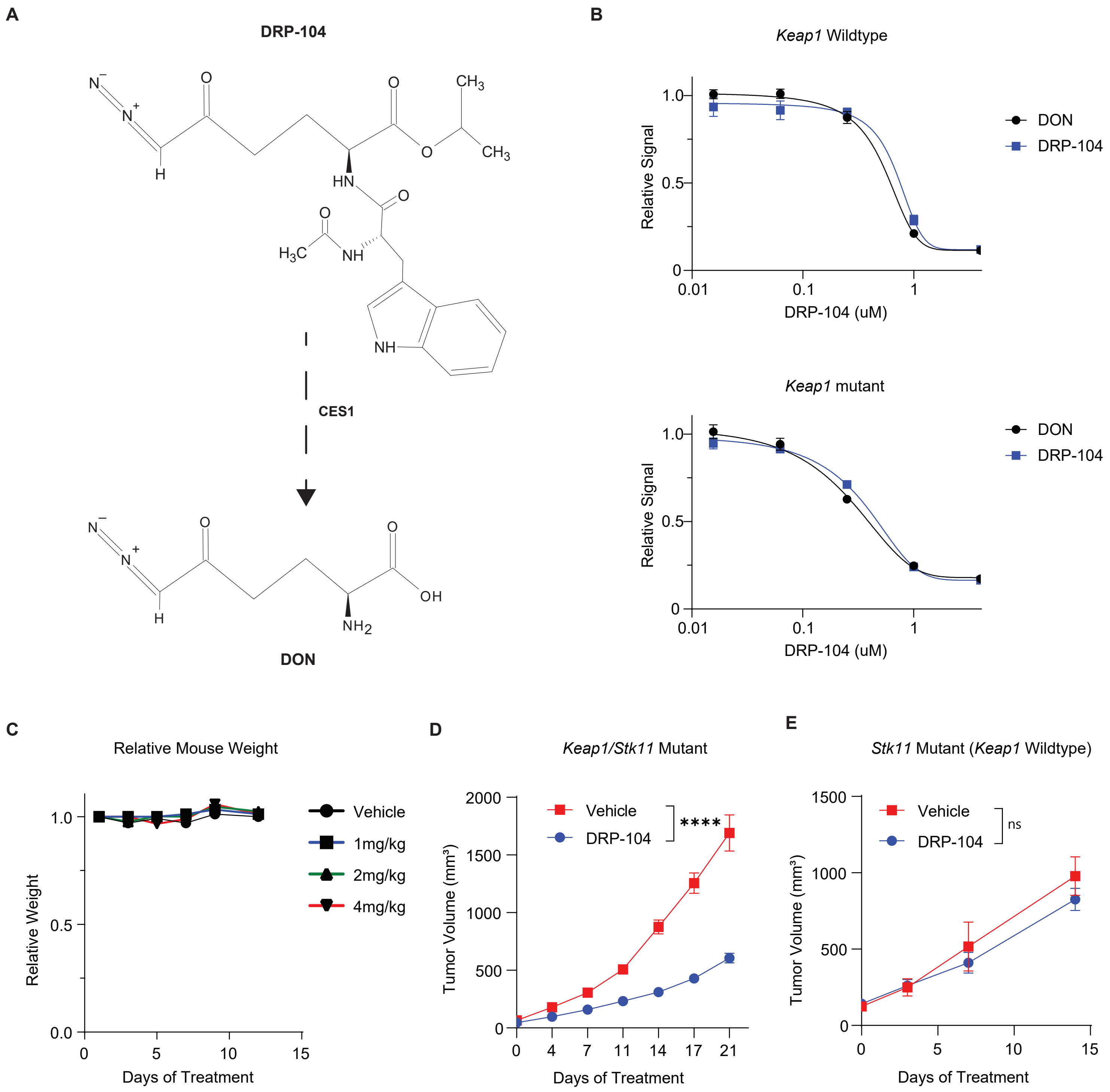

A
DRP-104
CES1
DON
B
Keap1 Wildtype
Relative Signal
DRP-104 (uM)
DON
DRP-104
Keap1 mutant
C
Relative Mouse Weight
Relative Weight
Days of Treatment
Vehicle
1mg/kg
2mg/kg
4mg/kg
D
Keap1/Stk11 Mutant
Tumor Volume (mm³)
Vehicle
DRP-104
****
E
Stk11 Mutant (Keap1 Wildtype)
ns

Figure S1: DRP-104 is effective *in vitro* and *in vivo* against *Keap1* mutant tumors
A: Structure of the prodrug DRP-104 which is enzymatically activated to 6-Diazo-6-oxo-l-norleucine (DON) by carboxylesterase 1 (CES1). **B**: *In vitro* dose response to DRP-104 and DON in *Keap1* wildtype and mutant cells. Cells were plated and treated with drug for three days and viability was measured by cell titer glo. **C**: C57BL/6 mice were treated with DRP-104 1, 2, or 4 mg/kg and relative weight to baseline is plotted. **D, E**: *Keap1/Stk11* co-mutant (**D**) or *Keap1* wildtype *Stk11* mutant (**E**) *Kras*$^{G12D/+}$ *p53*$^{-/-}$ cells were transplanted subcutaneously into C57BL/6 mice and response to DRP-104 (3 mg/kg) was measured (n=10 per group). Tumor growth was analyzed by two-way ANOVA. **** $p < 0.0001$, ns = not significant.

A

	Mutation Status				
**PDX**	***KRAS***	***p53***	***KEAP1***	***STK11***	**Histologic Subtype**
**TPX1**	-	-	WT	-	Adenocarcinoma
**CTG1194**	G12A	WT	WT	WT	Adenocarcinoma
**LX465**	G12D	L35Ffs*8	WT	WT	Adenocarcinoma
**LX326**	NRAS	R248L	G511C	WT	Adenocarcinoma
**LX55a**	G12C	R248L	D422N	WT	Adenocarcinoma
**LX337**	G12C	DeepDel	G332C	D53Tfs*11	Adenocarcinoma
**CTG743**	G12S	S215R	H311R	WT	Adenocarcinoma
**LX640**	WT	X261_splice	G511C	WT	Squamous

B

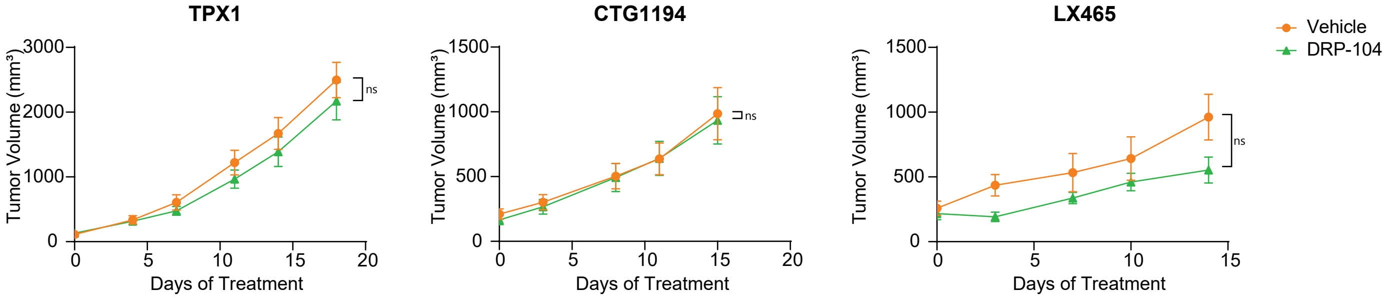

C

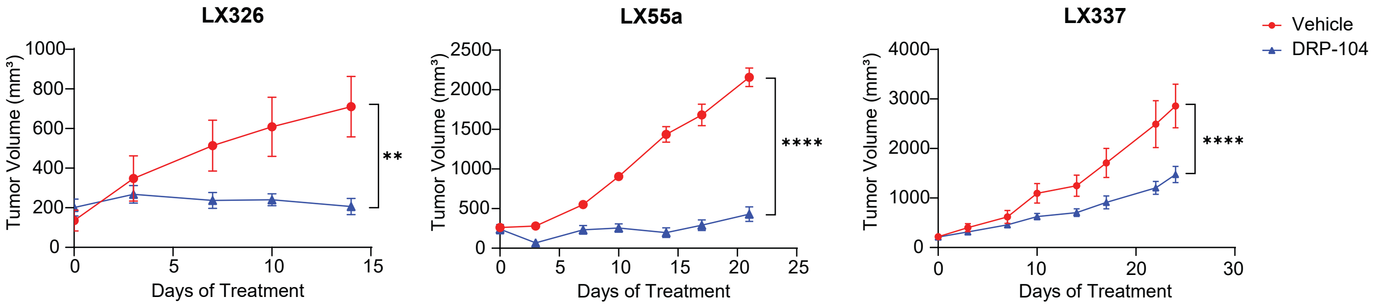

D

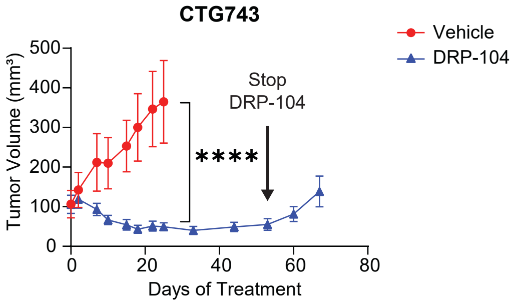

Figure S2: *KEAP1* mutant patient derived xenografts respond to DRP-104

A: Patient derived xenograft (PDX) lines with mutation status and histologic subtype listed. **B, C**: Treatment responses as measured by PDX tumor volume to DRP-104 (3 mg/kg) or vehicle control in **B**) *KEAP1* wildtype PDX (TPX1: n=8-10 per group, CTG1194: n=14-16 per group, LX465: n=8-9 per group) and **C**) *KEAP1* mutant PDX (LX326: n=6 per group, LX55a: n=8-9 per group, LX337: n=8 per group, (**D**) Treatment of *KEAP1* mutant PDX CTG743 with DRP-104 (3 mg/kg) or vehicle control (n=10 per group). Timepoint of withdrawal of DRP-104 is indicated. Tumor growth was analyzed by two-way ANOVA. ** $p<0.01$, **** $p < 0.0001$, ns= not significant.

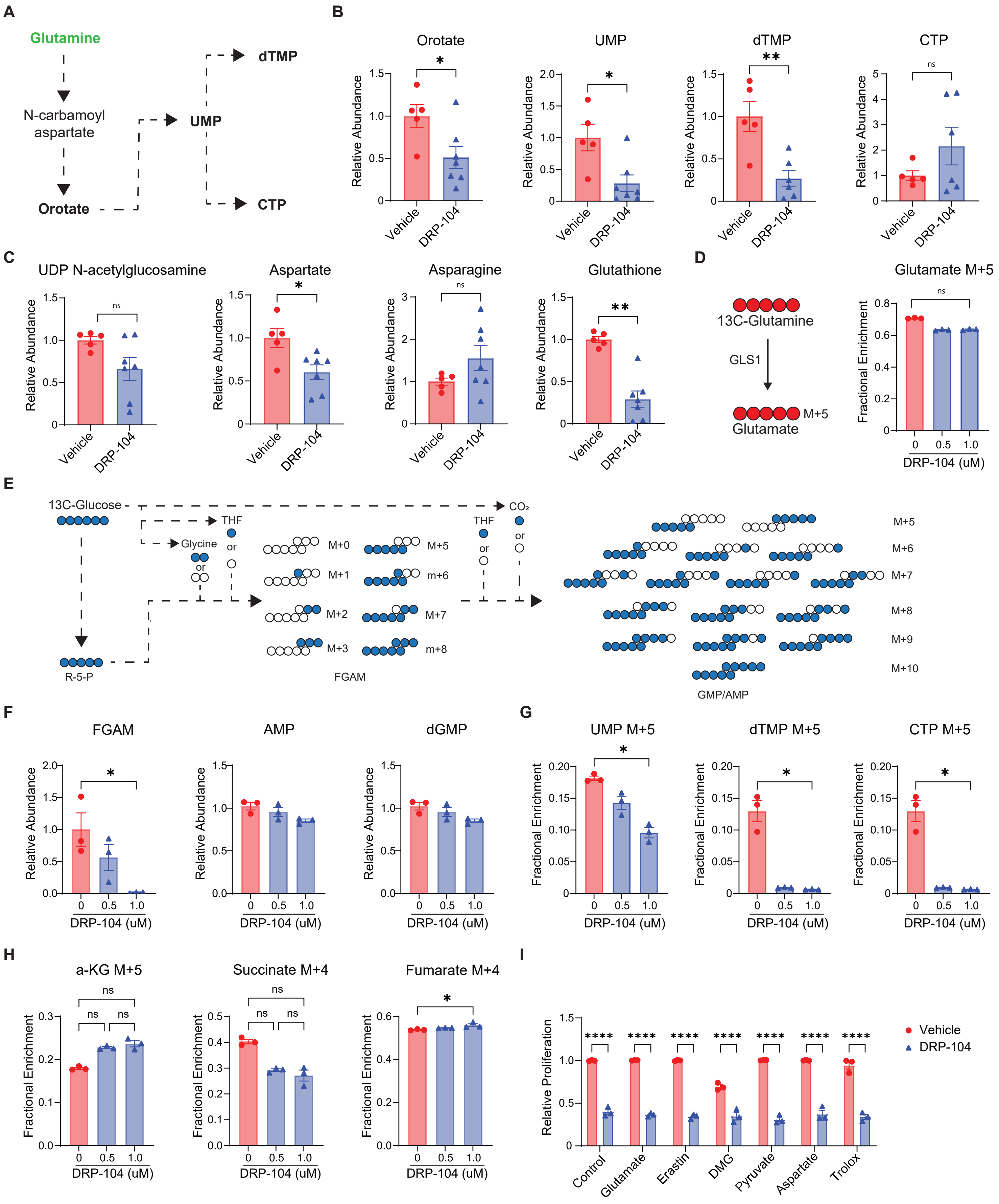

A
Glutamine
N-carbamoyl aspartate
Orotate
UMP
dTMP
CTP
B
Orotate
UMP
dTMP
CTP
Relative Abundance
Vehicle
DRP-104
C
UDP N-acetylglucosamine
Aspartate
Asparagine
Glutathione
D
13C-Glutamine
GLS1
M+5
Glutamate
Glutamate M+5
Fractional Enrichment
DRP-104 (uM)
E
13C-Glucose
THF
Glycine
or
CO_2
R-5-P
FGAM
GMP/AMP
M+0
M+1
M+2
M+3
M+5
m+6
M+7
m+8
M+6
M+8
M+9
M+10
F
FGAM
AMP
dGMP
G
UMP M+5
dTMP M+5
CTP M+5
H
a-KG M+5
Succinate M+4
Fumarate M+4
I
Relative Proliferation
Control
Glutamate
Erastin
DMG
Pyruvate
Aspartate
Trolox
Vehicle
DRP-104

Figure S3: *In vivo* and *in vitro* metabolomics of DRP-104 treated *KEAP1* mutant tumors

A: Overview of pyrimidine synthesis pathway. **B**, **C**: Levels of selected metabolites by liquid chromatography mass spectrometry in CTG743 (*Keap1* mutant) patient derived xenograft tumors after 5 days of DRP-104 treatment (n=5-7 per group). **D**: *In vitro* tracing of *Keap1* mutant tumor cells with DRP-104 (n=3 per group) after 1 hour of labeling with 13C-glutamine (left) and quantification of fractional enrichment of 13C-glutamine labeled glutamate for each concentration of DRP-104 (right). **E**: Schematic of 13C-Glucose tracing for purine synthesis. **F**: Relative abundance of FGAM, AMP, dGMP as measured by LCMS from *Keap1* mutant tumor cells measured *in vitro*. **G, H**: Fractional enrichment of pyrimidines (G) and the TCA intermediates a-KG, succinate, and fumarate (H) after 13C-Glucose labelling with DRP-104 treatment *in vitro* (n=3 per condition). **I**: *Keap1* mutant tumor cells were pretreated with specific metabolites 24 hours prior to DRP-104 treatment (2 uM). After five days of drug or vehicle treatment, proliferation was measured by crystal violet and plotted relative to controls (n=3 per condition). Statistical analysis was done by either Mann Whitney test, Kruskal-Wallis test with Dunn's multiple comparisons test, or 2-way ANOVA. ns = not significant, * $p < 0.05$, ** $p<0.01$, **** $p < 0.0001$.

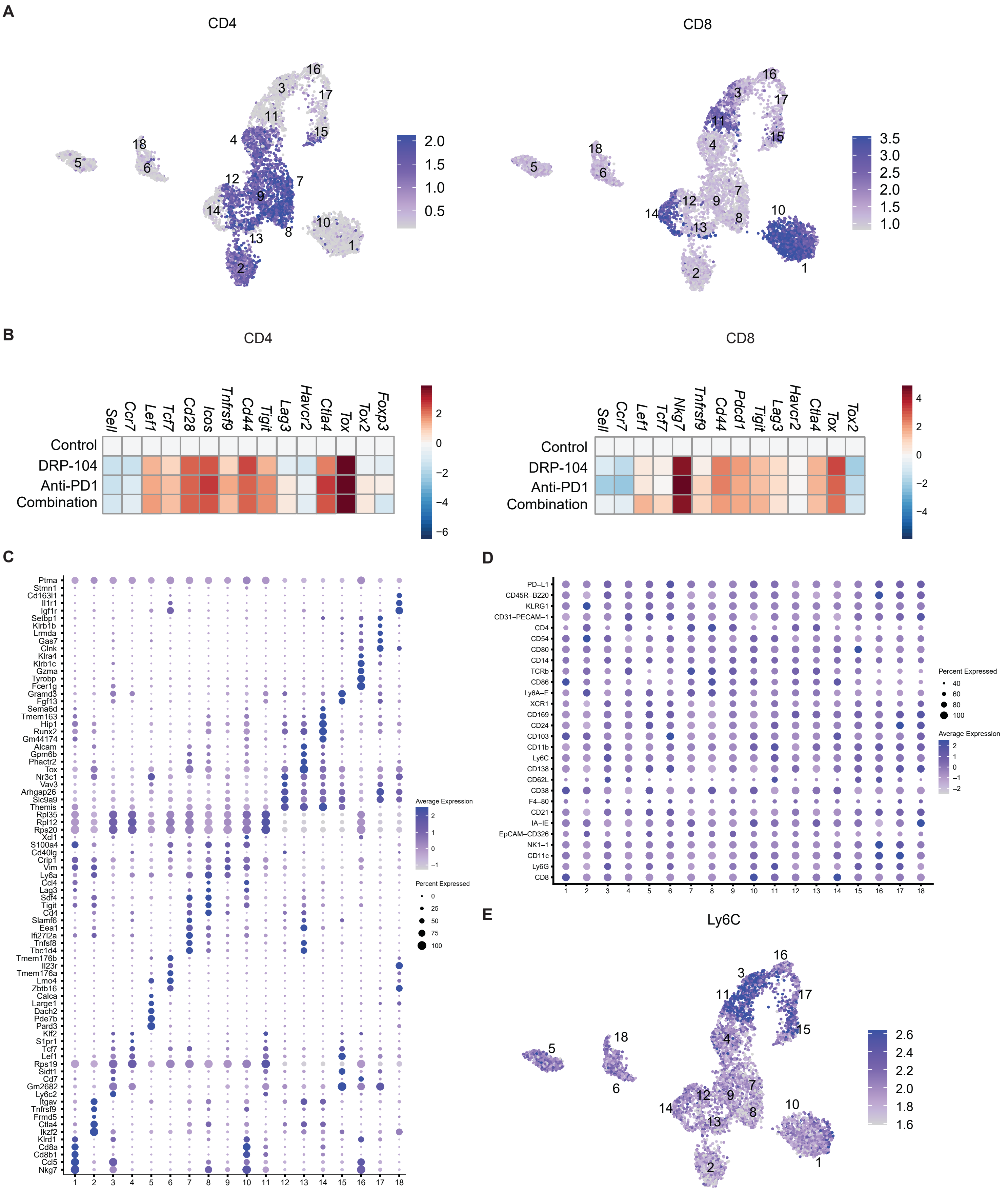

A
CD4
CD8
2.0
1.5
1.0
0.5
3.5
3.0
2.5
2.0
1.5
1.0
B
CD4
Sell Ccr7 Lef1 Tcf7 Cd28 Icos Tnfrsf9 Cd44 Tigit Lag3 Havcr2 Ctla4 Tox Tox2 Foxp3
Control
DRP-104
Anti-PD1
Combination
2
0
−2
−4
−6
CD8
Sell Ccr7 Lef1 Tcf7 Nkg7 Tnfrsf9 Cd44 Pdcd1 Tigit Lag3 Havcr2 Ctla4 Tox Tox2
Control
DRP-104
Anti-PD1
Combination
4
2
0
−2
−4
C
Ptma
Stmn1
Cd163l1
Il1r1
Igf1r
Setbp1
Klrb1b
Lrmda
Gas7
Clnk
Klra4
Klrb1c
Gzma
Tyrobp
Fcer1g
Gramd3
Fgf13
Sema6d
Tmem163
Hip1
Runx2
Gm44174
Alcam
Gpm6b
Phactr2
Tox
Nr3c1
Vav3
Arhgap26
Slc9a9
Themis
Rpl35
Rpl12
Rps20
Xcl1
S100a4
Cd40lg
Crip1
Vim
Ly6a
Ccl4
Lag3
Sdf4
Tigit
Cd4
Slamf6
Eea1
Ifi27l2a
Tnfsf8
Tbc1d4
Tmem176b
Il23r
Tmem176a
Lmo4
Zbtb16
Calca
Large1
Dach2
Pde7b
Pard3
Klf2
S1pr1
Tcf7
Lef1
Rps19
Sidt1
Cd7
Gm2682
Ly6c2
Itgav
Tnfrsf9
Frmd5
Ctla4
Ikzf2
Klrd1
Cd8a
Cd8b1
Ccl5
Nkg7
1 2 3 4 5 6 7 8 9 10 11 12 13 14 15 16 17 18
Average Expression
2
1
0
−1
Percent Expressed
0
25
50
75
100
D
PD–L1
CD45R–B220
KLRG1
CD31–PECAM–1
CD4
CD54
CD80
CD14
TCRb
CD86
Ly6A–E
XCR1
CD169
CD24
CD103
CD11b
Ly6C
CD138
CD62L
CD38
F4–80
CD21
IA–IE
EpCAM–CD326
NK1–1
CD11c
Ly6G
CD8
1 2 3 4 5 6 7 8 9 10 11 12 13 14 15 16 17 18
Percent Expressed
40
60
80
100
Average Expression
2
1
0
−1
−2
E
Ly6C
2.6
2.4
2.2
2.0
1.8
1.6

Figure S4: ExCITE-seq Analysis of T/NK/NKT/ILC Cells
A: Antibody derived tag (ADT) expression for CD4 and CD8 on T cell/NK cell/NKT cell/ILC subclusters. **B**: Heatmap for expression of selected genes in CD4 and CD8 T cells. **C**: Differentially expressed genes for T/NK/NKT/ILC subclusters. **D**: ADT expression by T cell/NK cell/NKT cell/ILC subcluster. **E**: Protein expression of Ly6C by ADT.

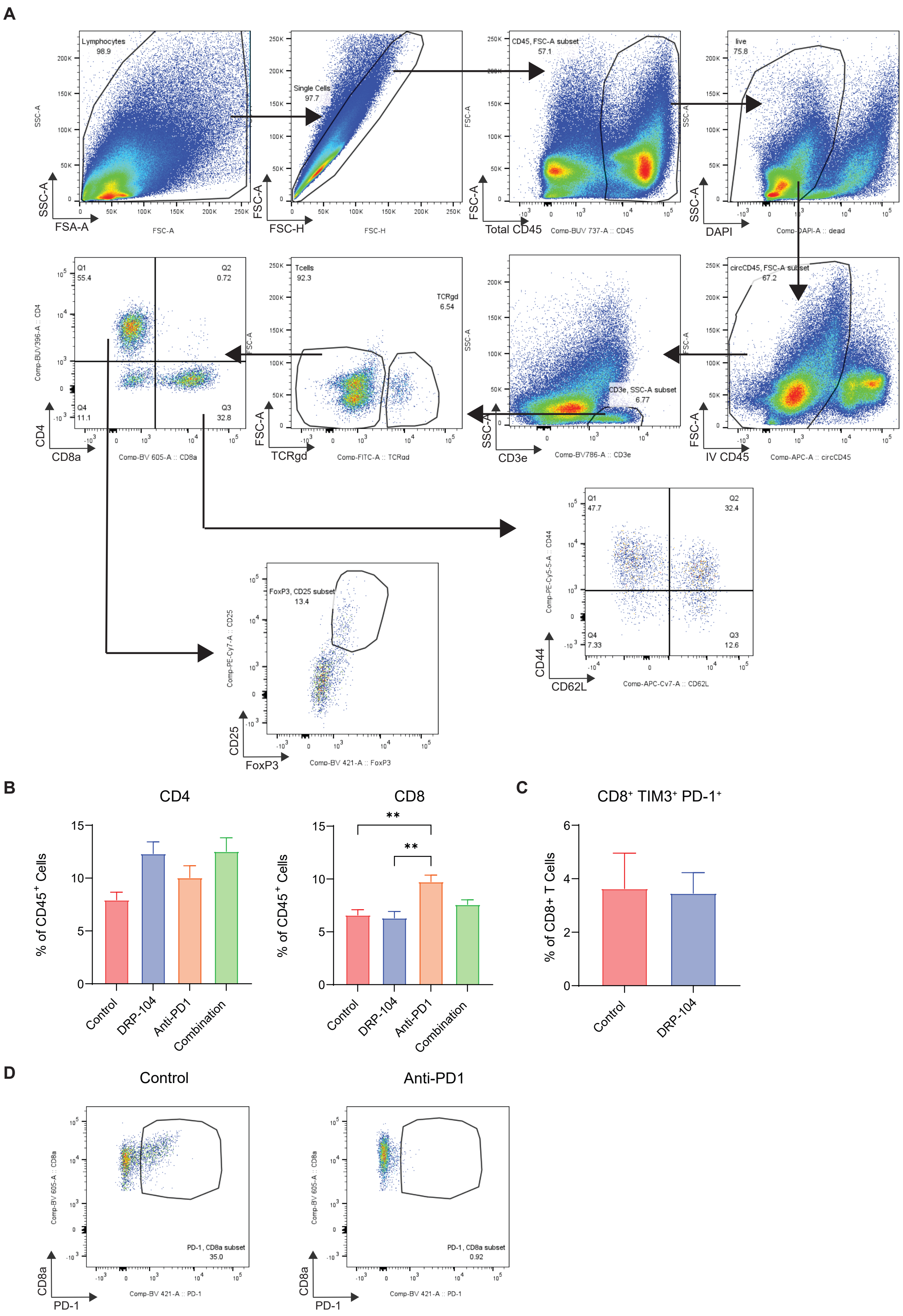
A
Lymphocytes
98.9
SSC-A
FSA-A
FSC-A
Single Cells
97.7
FSC-H
CD45, FSC-A subset
57.1
Total CD45
Comp-BUV 737-A :: CD45
live
75.8
DAPI
Comp-DAPI-A :: dead
circCD45, FSC-A subset
67.2
IV CD45
Comp-APC-A :: circCD45
CD3e, SSC-A subset
6.77
CD3e
Comp-BV786-A :: CD3e
Tcells
92.3
TCRgd
6.54
Comp-FITC-A :: TCRgd
Q1
55.4
Q2
0.72
Q4
11.1
Q3
32.8
CD4
CD8a
Comp-BUV396-A :: CD4
Comp-BV 605-A :: CD8a
Q1
47.7
Q2
32.4
Q4
7.33
Q3
12.6
CD44
CD62L
Comp-PE-Cy5-5-A :: CD44
Comp-APC-Cy7-A :: CD62L
FoxP3, CD25 subset
13.4
CD25
FoxP3
Comp-PE-Cy7-A :: CD25
Comp-BV 421-A :: FoxP3
B
CD4
% of CD45⁺ Cells
Control
DRP-104
Anti-PD1
Combination
CD8
**
**
C
CD8⁺ TIM3⁺ PD-1⁺
% of CD8+ T Cells
Control
DRP-104
D
Control
Anti-PD1
PD-1, CD8a subset
35.0
0.92
CD8a
PD-1
Comp-BV 605-A :: CD8a
Comp-BV 421-A :: PD-1

Figure S5: Flow Cytometry Analysis of T cells from orthotopic lung cancer model
A: Gating strategy for T cell populations. **B, C**: Flow cytometry quantification of (**B**) CD4, CD8, and (**C**) $CD8^+$ $TIM3^+$ $PD\text{-}1^+$ T cell populations of *Keap1* R470C mutant tumor bearing lungs after five days of DRP-104 (3 mg/kg daily) or vehicle and anti-PD1 (200 ug three times a week) or isotype control treatment (n=5 per group). **D**: Representative flow cytometry plots of $PD\text{-}1^+$ expression in CD8 T cells isolated from Keap1 R470C mutant tumor bearing lungs after treatment with anti-PD1 or isotype control. Statistical analysis was done by one-way ANOVA with Tukey's multiple comparisons test or Mann Whitney test. ** $p<0.01$

Supplementary Table 1

Antigen	Clone	Manufacturer
CD45	30-F11	Biolegend
CD45	30-F11	BD Biosciences
CD3e	17A2	Biolegend
CD4	GL15	BD Biosciences
CD4	RM4-5	Invitrogen
CD8a	53-6.7	Biolegend
CD8a	53-6.7	BD Biosciences
TCRgd	UC7-13D5	Biolegend
CD62L	MEL-14	eBioscience
CD44	IM7	eBioscience
FoxP3	FJK-16S	eBioscience
LAG3	C9B7W	eBioscience
TIM3	RMT3-23	Biolegend
TCF7	C63D9	Cell Signaling Technologies
IFNγ	XMG1.2	BD Biosciences
TNFα	MP6-XT22	eBioscience
PD-1	RMP1-30	eBioscience
CD25	PC61.5	eBioscience

Supplementary Table 1: Antibody List
